# Synthesis of a bifunctional cytidine derivative and its conjugation to RNA for in vitro selection of a cytidine deaminase ribozyme

**DOI:** 10.3762/bjoc.10.198

**Published:** 2014-08-15

**Authors:** Nico Rublack, Sabine Müller

**Affiliations:** 1 Institut für Biochemie, Ernst-Moritz-Arndt-Universität Greifswald, Felix-Hausdorff-Str. 4, D-17487 Greifswald, Germany

**Keywords:** cytidine deaminase, modified nucleoside, nucleic acids, ribozyme, RNA world, SELEX

## Abstract

Over the past 20 years, the generation of functional RNAs by in vitro selection has become a standard technique. Apart from aptamers for simple binding of defined ligands, also RNAs for catalysis of chemical reactions have been selected. In the latter case, a key step often is the conjugation of one of the two reactants to the library, requiring suitable strategies for terminal or internal RNA functionalization. With the aim of selecting a ribozyme for deamination of cytidine, we have set up a selection scheme involving the attachment of the cytidine acting as deamination substrate to the 3'-terminus of the RNAs in the library, and library immobilization. Here, we report the synthesis of a bifunctional cytidine derivative suitable for conjugation to RNA and linkage of the conjugated library to a streptavidine-coated surface. Successful conjugation of the cytidine derivative to the 3'-terminus of a model RNA is demonstrated.

## Introduction

Since the discovery of the first catalytic RNA in the ciliate *Tetrahymena thermophilia* in 1982 [1], a number of naturally occurring ribozymes have been described [2]. Whereas all of these natural ribozymes accelerate transesterifications or, as in the case of the ribosome, the peptide bond formation, artificial ribonucleic acids developed by in vitro selection have been shown to catalyze a wide variety of organic chemical reactions [3–5]. Moreover, several of these developed ribozymes promote reactions of metabolic relevance in modern life organisms. Impressive examples are RNA catalysts that support an aldol reaction between an aldehyde and a ketone, relevant to the synthesis of sugars [6], or the linkage of ribose to nucleobases to generate nucleotides [7]. Such ribozymes are seen as important functional entities underscoring the RNA world theory, where ribonucleic acids are suggested acting as the carrier of genetic information as well as functional players enabling RNA amplification and processing [8]. Even though a number of ribozymes supporting the RNA world theory are known, there is a wide field of protein-catalyzed reactions in present-day organisms, for which no ribozyme analogue has yet been found. This applies for example to the transformation of cytidine to uridine, which is a well-known RNA editing event in modern cellular chemistry [9]. This process is catalyzed by cytidine deaminases (CDA, EC 3.5.4) belonging to a family of enzymes found in pro- and eukaryotes [10]. In the active center of all CDAs, a zinc ion is responsible for the activation of a water molecule that acts as nucleophile attacking the C4 carbon center of the cytosine residue and thus facilitating deamination [11]. The development of a ribozyme supporting the same kind of reaction would be a valuable addition to the repertoire of RNA activities with relevance to the RNA world theory.

A very useful and often applied technique for the generation of catalytic RNA structures is a variation of the classical SELEX approach (Systematic Evolution of Ligands by EXponential Enrichment) [12–13]. A typical procedure for the selection of a ribozyme, which enhances the reaction between two substrates, involves the conjunction of either of them with the members of an RNA-library. This is mostly achieved by transcription priming [14], which however, allows attachment of a specific reactant merely to the 5'-terminus of library RNAs [15–17]. Alternatively, post-transcriptional protocols can address both the 5'- and the 3'-terminus. In general, post-transcriptional 5'-modification may be achieved by thiophosphorylation with T4-polynucleotide kinase followed by derivatization of the introduced terminal thiophosphate [18–19], or by chemical conversion of the 5'-terminal primary OH group into an amine or azide to be used for further conjugation with NHS-esters [20] or with alkynes [21]. Alternatively, natural and modified nucleosides can be attached to the 3'-terminus by the use of enzymes like poly(U)-polymerase (PUP), poly(A)-polymerase (PAP) or the terminal deoxynucleotidyl transferase (TdT) [22–25]. However, since most enzymatic techniques achieve only moderate yields, chemical strategies may be advantageous. Chemical 3'-end modification uses the unique properties of the RNA's 3'-terminal *cis*-diol, which can be specifically oxidized with metaperiodate [26–28], followed by reaction of the produced dialdehyde with amines or hydrazines linked to a desired functional entity [29].

**Figure 1:**
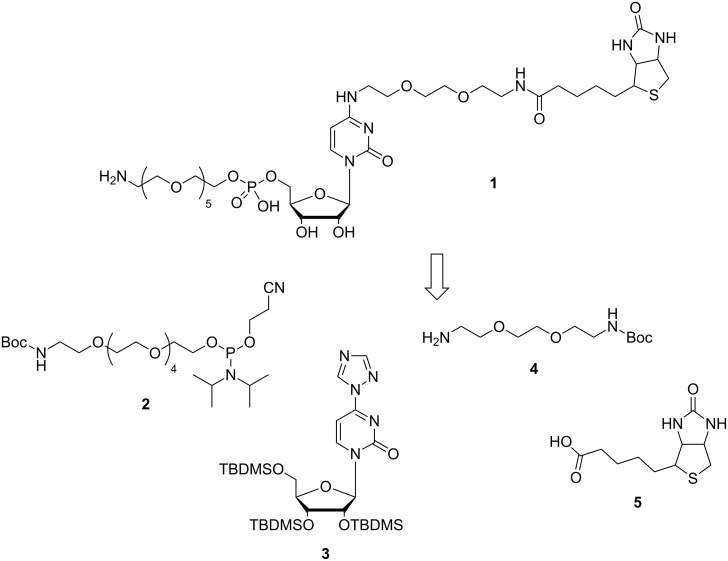
Retrosynthetic analysis of the bifunctional cytidine derivative **1** for functionalization of a periodate-oxidized RNA library.

We set out to select from a random library catalytically active RNAs that support deamination of cytidine to uridine. For this purpose, a bifunctionalized cytidine derivative (Figure 1) was synthesized. Via its 5'-OH group, the cytidine derivative is linked to a hexaethylene glycol tether bearing a primary amino group. At the C4-position of the base, a short linker connected to a biotin was introduced. Upon periodate oxidation of the 3'-*cis* diol of the RNA library molecules, the produced dialdehyde is thought to react with the primary amino group of the cytidine derivative allowing immobilization of the resulting modified RNAs onto a solid surface through biotin–streptavidine interaction. All RNA sequences that can catalyze the desired deamination reaction of cytidine to uridine will be cleaved off and thus released into solution. Upon recovery, beneficial variants can be subjected to the next round of selection. Here we describe the synthetic route to the bifunctionalized cytidine derivative and its successful conjugation to RNA.

## Results and Discussion

Synthesis of the bifunctional cytidine derivative **1** started from uridine making use of four synthons: a protected hexaethylene glycol linker phosphoramidite **2** bearing a primary amine to be used later for RNA functionalization, protected and suitably activated uridine **3**, and a short mono protected diamino linker **4** for the attachment of a biotin moiety **5** in order to immobilize the conjugated RNA molecules onto a streptavidine-coated surface (Figure 1).

**Figure 2:**
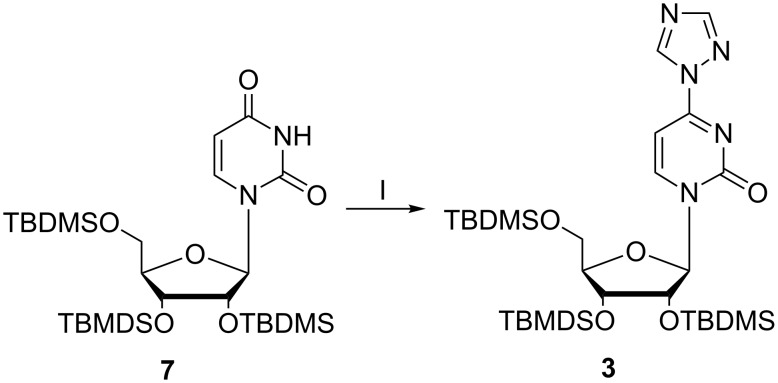
Introduction of the triazolyl moiety into the uridine derivative **7** generating synthon **3**. I: 4 equiv POCl_3_, 16 equiv triazole, 20 equiv NEt_3_, MeCN, 60 min at 0 °C, 4.5 h at rt, 76%.

First, the sugar hydroxy functions of uridine (**6**) were fully protected with *tert*-butyldimethylsilyl (TBDMS) groups and the tris-silylated uridine **7** [30] was further reacted with POCl_3_, triazole and triethylamine (Figure 2).

**Figure 3:**
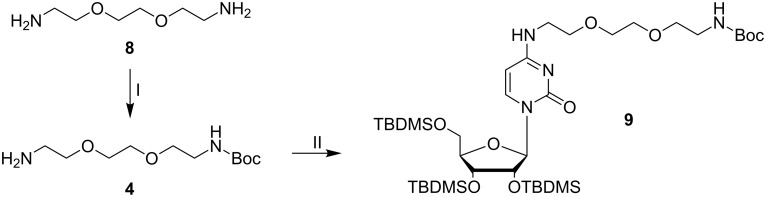
Preparation of synthon **4** and substitution of the triazolyl moiety of **3** to form the fully protected cytidine derivative **9**. I: 0.16 equiv Boc_2_O, dioxane, 3 h, rt, 68% (based on the amount of Boc_2_O); II: 0.77 equiv triazolyl protected uridine **3**, MeCN, rt, overnight, 85%.

Among the procedures described for conversion of uridine into cytidine derivatives [31–34] we have chosen the path via a triazolyl activated uridine [31], because the triazolyl derivatives are known being stable enough to allow convenient handling and isolation, and they have shown very good reactivity with aliphatic amines. Synthon **3** could be isolated by recrystallization in 76% yield. Next, we introduced a short linker to the N^4^-position of **3** in order to attach a biotin unit to the modified nucleoside. Linker **4** was synthesized from 2,2’-(ethylenedioxy)diethylamine (**8**) with di-*tert*-butyl dicarbonate (Boc_2_O) in dioxane [35] (Figure 3).

The mono protected diamino linker **4** was purified by chromatography on silica gel and subsequently used for reaction with the triazolyl protected uridine derivative **3** to produce compound **9** in 85% yield. Upon removal of the Boc group and sugar deprotection, the remaining aliphatic amine should be used for conjugation to biotin, followed by coupling of the 5'-hydroxy group to a linker phosphoramidite.

**Figure 4:**
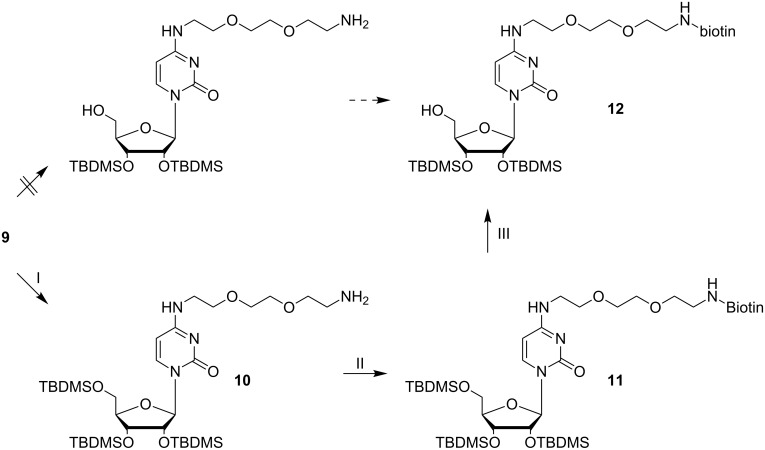
Synthesis of 2',3'-bis-*O*-(*tert*-butyldimethylsilyl)-1-[4-(*N*'-biotinyl-3,6-dioxaoctane-1,8-diamine)pyrimidine-2(1*H*)-onyl]-β-D-riboside (**12**). I: 2.6 equiv ZnBr_2_, DCM, 1 d, rt, Ar, 82%; II: 1.1 equiv EDAC·HCl, 1.1 equiv biotin, DMF, 0 °C → rt, overnight, 65%; III: THF/TFA/H_2_O (4:1:1, v/v/v), 0 °C, 5 h, 94%.

In terms of synthesis efficiency, we first tried to simultaneously deprotect the amino functionality at the nucleobase and the sugar 5'-hydroxy group to generate derivative **12**. Thereafter, the greater nucleophilicity of the primary amine over the alcohol should be used for selective amide bond formation with biotin, provided for reaction as *N*-hydroxysuccinimidyl (NHS-) ester, and subsequently the 5'-*O*-phosphoramidite should be prepared. Unfortunately, preparation of **12** in the suggested one step procedure (Figure 4, upper path) was not possible.

**Figure 5:**
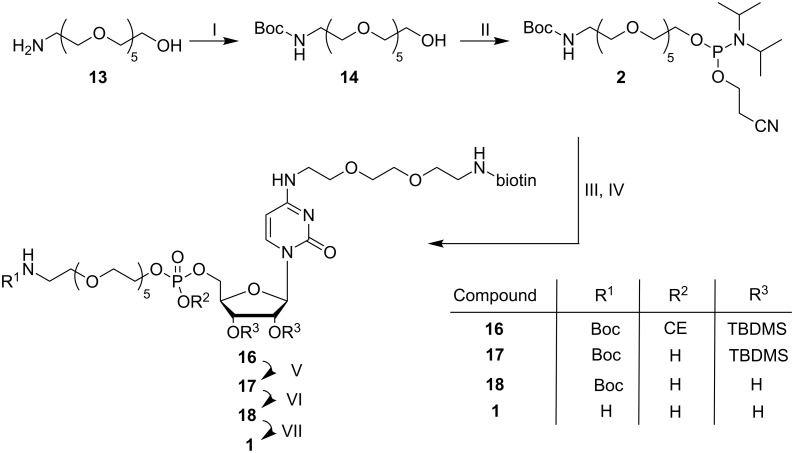
Formation of the phosphoramidite **2** from amino alcohol **13**, and subsequent coupling to the 5'-*O*-deprotected nucleoside **12**. I: 1.2 equiv di-*tert*-butyl dicarbonate, EtOAc, 60 °C, 1.5 h, 98%; II: 5.3 equiv DIPEA, 1.3 equiv (2-cyanoethyl-*N*,*N*-diisopropyl)chlorophosphoramidite, DCM, rt, 1.5 h; III: 0.9 equiv **12**, 4.5 equiv BMT, THF, IV: 0.2 M I_2_ in THF/pyridine/H_2_O, 10 min, rt, 28% over two steps; V: aq NH_3_ (30%)/methylamine (8 M), rt, 30 min; VI: TEA·3HF, 55 °C, 2 h; VII: TFA, rt, 2 min; 83 % over three steps.

According to a previous report by Zhu et al. [36], the 5'-OH group of tris-silylated nucleosides can be selectively removed by treatment with a THF/TFA/H_2_O mix (4:1:1, v/v/v) at 0 °C. Since cleavage of Boc-groups requires strong Brønsted acids [37–39], we varied the protocol of Zhu et al. with respect to the acid concentration and to reaction time. However, we could not identify conditions that led to removal of the Boc group, and leaving the TBDMS groups intact; multiple desilylated products were detected in all cases. These findings forced us to synthesize **12** in an alternative way. Apart from protic acids, the *tert*-butoxycarbonyl moiety of Boc-protected amines can be selectively cleaved with Lewis acids [40]. Thus, stirring of **9** in anhydrous DCM with ZnBr_2_ delivered the free amine **10** with a yield of 82% (Figure 4). To this, biotin was coupled by in situ activation of the carboxylic acid as an NHS-ester using 1-ethyl-3-(3-dimethylaminopropyl)carbodiimide hydrochloride (EDAC·HCl). Next, the 5'-*O*-TBDMS group was removed by stirring **11** in a mixture of THF/TFA/H_2_O (4:1:1, v/v/v) at 0 °C, and solely the 5'-*O*-deprotected cytidine derivative **12** was obtained in 94% yield (Figure 4). The long amino linker **13** was synthesized from hexaethylene glycol with an overall yield of 52% over three steps following a standard literature protocol [41]. After introduction of a Boc group for protection of the linker amino function, the resulting compound **14** was reacted with (2-cyanoethyl-*N*,*N*-diisopropyl)chlorophosphoramidite to yield the phosphoramidite **2**, which was coupled with synthon **12** in anhydrous THF in the presence of 5-benzylmercapto-1*H*-tetrazole (BMT) as activator (Figure 5).

**Figure 6:**
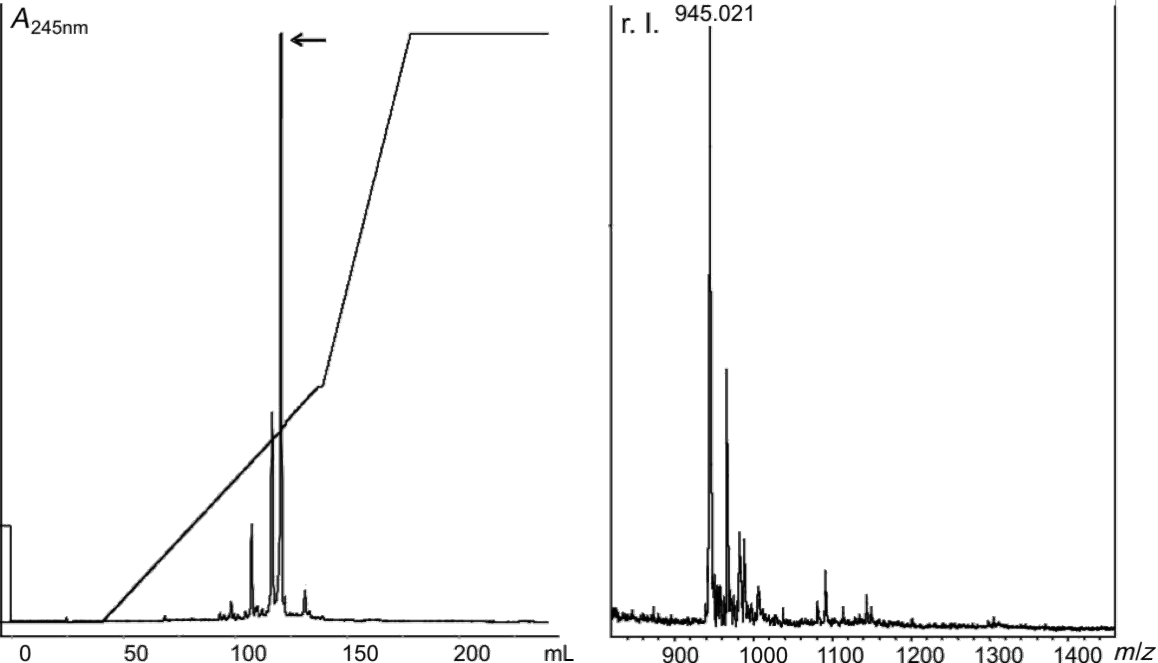
A) Reversed-phase HPLC purification of **1** after complete deprotection of **16. ***A* represents the absorption at 254 nm. The fraction indicated by an arrow was collected and analyzed by MALDI mass spectrometry. B) MALDI-spectrum of the pooled collected fractions. The peak at 945.021 (calcd. mass: 944.40 g/mol [M + H]^+^) corresponds to the cytidine derivative **1**. r.I. represents the relative intensity.

The coupling product **16** was isolated with a yield of 28%. Finally, all remaining protecting groups were cleaved off. The base-labile β-cyanoethyl (CE) group was removed by stirring **16** in a 1:1 mixture of concentrated aqueous ammonia and methylamine. Subsequent treatment of **17** with triethylamine trihydrofluoride (TEA·3HF) at 55 °C for 2 h delivered compound **18**, which was reacted with neat TFA. Each deprotection step was monitored by MALDI mass spectrometry. The bifunctionalized cytidine derivative **1** as the final product was isolated by reversed-phase chromatography with a yield of 83% over the last three steps (Figure 6).

**Figure 7:**
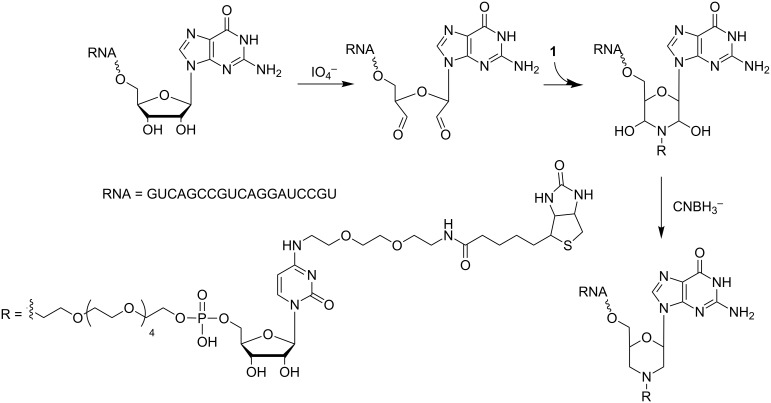
Reaction scheme of periodate oxidation of a 20mer model RNA followed by coupling of cytidine derivative **1**.

To set-up conditions for conjugation of the bifunctional cytidine derivative **1** to RNA, we synthesized a short model oligonucleotide (GUC AGC CGU CAG GAU CCG UG) corresponding to the 3'-terminal sequence of the envisaged RNA-library. The RNA was oxidized by treatment with sodium periodate [42], and the resulting dialdehyde was reacted with cytidine derivative **1**. The obtained intermediate was reduced with sodium cyanoborohydride to form the more stable amine as final product (Figure 7).

**Figure 8:**
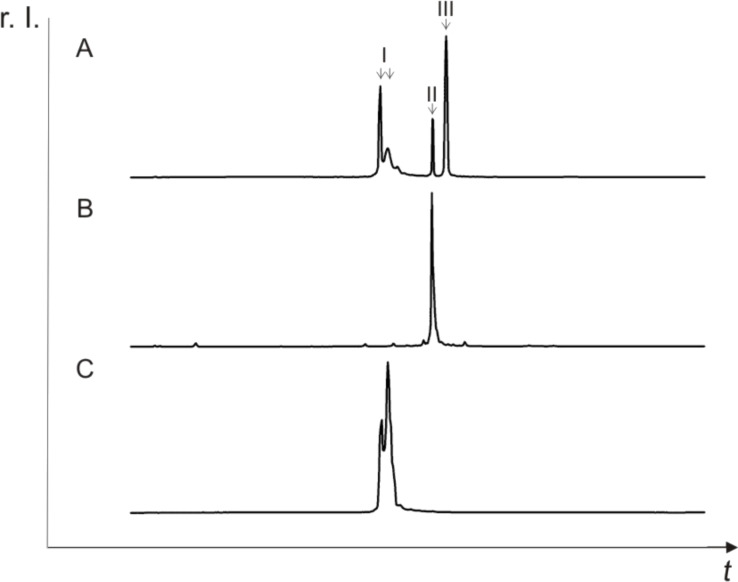
Reversed-phase HPLC analysis. A: Crude product of the coupling reaction between the 20mer model RNA and **1.** The peaks I correspond to the periodate oxidized and subsequently reduced RNA. Peak II matches the cytidine derivative **1**. Peak III indicates the RNA–cytidine **1** conjugate. B: Cytidine derivative **1**. C: 20mer model RNA after treatment with sodium periodate and reduction with sodium cyanoborohydride in the absence of cytidine derivative **1**. r. I.: relative intensity.

The reaction analysis was carried out by reversed-phase HPLC (Figure 8).

The RP-chromatogram of the crude product (A) shows two peaks (denoted as I) corresponding to the oxidized and cyanoborohydride reduced RNA (C), peak II corresponding to the cytidine derivative **1** (B), and a new peak (III) indicating the desired RNA–cytidine conjugate, as confirmed by MALDI mass spectrometry (see Supporting Information File 1).

## Conclusion

We successfully synthesized a cytidine derivative for the functionalization of an RNA library following a synthetic route over 16 steps. Two linker moieties of different lengths and functionality were attached to the nucleobase and to the sugar residue of cytidine, thus enabling for one coupling of the nucleoside to periodate-oxidized RNAs, and for second linking the functionalized library to a surface. The 5'-*O*-coupled linker carrying an aliphatic amino group allows 3'-terminal conjugation of cytidine to the molecules of an RNA library, whereas the biotinylated linker attached to C4 of the nucleobase allows linking the modified library to a streptavidine coated surface. The suitability of the synthesized cytidine derivative was confirmed by successful conjugation to the 3'-terminus of a model RNA, as analyzed by HPLC and MALDI–MS. This set-up is going to be used for selection of a cytidine deaminase ribozyme supporting the conversion of uridine to cytidine. Active molecules will be cleaved from the solid phase and released into solution, such that those can be collected, reverse-transcribed and amplified to enter the next round of selection.

## Experimental

The bifunctional cytidine derivative **1** was synthesized starting from uridine. All reaction steps and the characterization of the obtained products are described in detail in Supporting Information File 1. The RNA GUC AGC CGU CAG GAU CCG UG used as model for studying 3'-terminal conjugation of **1** was synthesized on an Applied Biosystems 394 DNA/RNA Synthesizer following the standard protocol for oligoribonucleotide chain assembly. The synthesized RNA was deprotected using aqueous ammonia (32%)/aq methylamine (40%) (1:1, v/v) at 65 °C for 30 min for removal of base and phosphate protecting groups and cleavage from the support, and TEA·3HF for removal of 2'-*O*-protecting groups, essentially as described previously [43].

### Periodate oxidation

For the oxidation of the RNA's 3'-terminal *cis*-diol, 5 nmol of the 20mer RNA substrate were incubated in a total volume of 1 mL 30 mM sodium metaperiodate, 100 mM NaOAc (pH 5.4) for 1 hour at room temperature in the dark. The oxidized RNA was recovered by ethanol precipitation.

### Conjugation of the cytidine derivative **1** to RNA

The oxidized RNA was dissolved in 250 µL water containing 20 mM imidazole (pH 8), 5 mM NaCNBH_3_, 1 mM EDTA and 1 mM cytidine derivative **1**. The reaction was carried out at 37 °C. After 2 hours, 25 µL of 50 mM NaBH_4_ were added, and the reaction mixture was incubated for additional 15 min. Ethanol precipitation yielded the crude coupling product, which was analyzed by reversed-phase HPLC on an Äkta Purifier (Amersham Bioscience). Column: Macherey Nagel EC 250/4 Nucleodur 100-5 C18 ec; Buffers: (A) 0.1 M triethylammonium acetate (pH 7.0), 5% acetonitrile and (B) 0.1 M triethylammonium acetate (pH 7.0), 30% acetonitrile; flow rate 0.5 mL min^−1^; gradient: 0% → 85% (B) in 14 CV, 85% 4 CV, 85% → 100% (B), 100% (B) 4 CV, 100% → 0% (B) 2 CV. The product containing fraction was collected, RNA was lyophilized and analyzed by MALDI–MS: (calcd mass: 7339.9 g/mol, found: 7339.04 [M + 1]^+^; 3668.515 [M + 2]^+^/2, Supporting Information File 1).

## Supporting Information

File 1 Experimental part.
