# Supplementary material for: Synthesis of a bifunctional cytidine derivative and its conjugation to RNA for in vitro selection of a cytidine deaminase ribozyme
Source: Beilstein J Org Chem. 2014 Aug 15;10:1906–13. doi: 10.3762/bjoc.10.198 (PMC4168874; doi:10.3762/bjoc.10.198)

Supporting Information

for

Synthesis of a bifunctional cytidine derivative and its conjugation to RNA for in vitro selection of a cytidine deaminase ribozyme

Nico Rublack and Sabine Müller*

Address: Institut für Biochemie, Ernst-Moritz-Arndt-Universität Greifswald, Felix-Hausdorff-Str. 4, D-17487 Greifswald, Germany

Email: Sabine Müller* - smueller@uni-greifswald.de

*Corresponding author

Experimental part

List of contents

General remarks…………………….……………………………………….………….S3

Synthesis of 2',3',5'-tris-*O*-(*tert*-butyldimethylsilyl)-1-[4-triazolyl-pyrimidine-2(1*H*)-onyl]-β-D-riboside (**3**)……………………….………………………………….S4

Synthesis of *N*-(*tert*-butoxycarbonyl)-3,6-dioxaoctan-1,8-diamine (**4**)………………S6

Synthesis of 2',3',5'-tris-*O*-(*tert*-butyldimethylsilyl)-1-[4-(*N*'-(*tert*-butoxycarbonyl)-3,6-dioxaoctane-1,8-diamine)pyrimidine-2(1*H*)-onyl]-*β*-D-riboside (**9**)…….….…...S8

Synthesis of 2',3',5'-tris-*O*-(*tert*-butyldimethylsilyl)-1-[4-(3,6-dioxaoctane-1,8-diamine)-pyrimidine-2(1*H*)-onyl]-β-D-riboside (**10**)……………………………………………S10

Synthesis of 2',3',5'-tris-*O*-(*tert*-butyldimethylsilyl)-1-[4-*N*'-biotinyl-3,6-dioxaoctane-1,8-diaminepyrimidine-2(1*H*)-onyl]-*β*-D-riboside (**11**)…………………………………S12

Synthesis of 2',3'-bis-*O*-(*tert*-butyldimethylsilyl)-1-[4-*N*'-biotinyl-3,6-dioxaoctane-1,8-diaminepyrimidine-2(1*H*)-onyl]-β-D-riboside (**12**)………………………………...S14

Synthesis of*N*-(*tert*-butoxycarbonyl)-17-amino-3,6,9,12,15-pentaoxaheptadecane-1-ol (**14**)……………………………………………………………………………....S16

Synthesis of *N*-(*tert*-butoxycarbonyl)-hexaethyleneglycolmonoaminephosphor-amidite (**2**)...S18

Synthesis of N^4-(3,6-dioxa-8-*N*'-biotinyl)-2',3'-bis-*O*-(*tert*-butyldimethylsilyl)-5'-*O*-β-cyanoethylphosphoryl-[(*N*-*tert*-butoxycarbonyl)-17-amino-3,6,9,12,15-penta-oxaheptadecyl]cytidine (**16**)……………………………………………..…………S20

Synthesis of N^4-(3,6-Dioxa-8-*N*'-biotinyl)-5'-*O*-phosphoryl-(17-amino-3,6,9,12,15-pentaoxaheptadecyl)cytidine (**19**)……………………………………..……...S22

MALDI-Tof analysis of RNA-cytidine **1** adduct…………….………………………...S24

General remarks

Proton nuclear magnetic resonance spectra (^{1}H NMR, 300 MHz) and proton-decoupled carbon nuclear magnetic resonance spectra (^{13}C NMR, 75 MHz) were obtained in deuterated chloroform ($CDCl_3$) and D_2O,with residual protonated solvent as internal standard. For ^{31}P NMR, chemical shifts were referenced to 85% phosphoric acid (0.0 ppm). Abbreviations for multiplicity are s, singlet; d, doublet; t, triplet; m, multiplet; br, broad. Mass spectra were recorded on a Bruker microflex MALDI–TOF MS. HPLC spectra were performed on an Äkta Purifier (Amersham Biosciences) with the columns described.

Diisopropylethylamine (DIPEA) was stored over calcium hydride and distilled before use. All other reagents, chemicals, buffers and solvents were obtained as the highest commercially available grade and used without further purification. Silica gel for column chromatography (0.063–0.2 mm) was obtained from Sigma-Aldrich. TLC chromatography was performed on pre-coated aluminium silica gel 60 F254 plates (Merck), and silica gel 60 (Macherey-Nagel) was used for column chromatography.

.

Synthesis of 2',3',5'-tris-*O*-(*tert*-butyldimethylsilyl)-1-[4-triazolylpyrimidine-2(1H)-onyl]-β-D-riboside (3)

A suspension of triazol (7.67 g, 111 mmol) in absolute acetonitrile was chilled in an ice-bath. $POCl_3$ (2.56 mL, 28 mmol) was added dropwise, followed by the addition of triethylamine (19.5 mL, 140 mmol). The suspension was stirred at 0 °C for 1 h. Compound **7** (4.10 g, 7 mmol) was added and the ice-bath removed. After stirring at rt for 4.5 h, the reaction mixture was cooled to 0 °C and quenched with $NaHCO_3$ (40 mL). After 10 min at rt, the solvents were removed under diminished pressure. The residue was dissolved in DCM (200 mL), and the solution was washed two times with saturated aq. $NaHCO_3$ (75 mL each) and saturated aq. NaCl (75 mL each), dried over Na_2SO_4,filtered and concentrated under reduced pressure. Recrystallization from ethanol gave compound **3** (3.39 g, 76%) as colorless crystals.^{1}H NMR (300 MHz, $CDCl_3$) δ (ppm) 9.22 (s, 1 H, triazol-C*H*), 8.84 (d, *J* = 7.2 Hz, 1 H, 6-C*H*), 8.08 (s, 1 H, triazol-C*H*), 6.91 (d, *J* = 7.2 Hz, 1 H, 5-C*H*), 5.75 (s, 1 H, 1'-C*H*), 4.18-4.10 (m, 3 H, 2'-C*H*, 3'-C*H*, 4'-C*H*), 3.99-4.03 (m, 1 H, 5'-C*H*), 3.79 (m, 1 H, 5''-C*H*), 0.95 (s, 9 H, Si-C(C*H*3)3), 0.89 (s, 9 H, Si-C(CH_3)$_3$), 0.84 (s, 9 H, Si-C(CH_3)$_3$), 0.25 (s, 3 H, Si-(CH_3)$_2$), 0.15 (s, 3 H, Si-(CH_3)$_2$), 0,14 (s, 3 H, Si-(CH_3)$_2$), 0.11 (s, 6 H, Si-(CH_3)$_2$), 0.08 (s, 3 H, Si-(CH_3)$_2$); ^{13}C NMR (300 MHz, $CDCl_3$) δ (ppm) 159.21 (2-*C*O), 154.43 (triazol), 153.95 (4-*C*N), 147.31 (6-*C*H), 143.24 (triazol), 93,86 (5-*C*H), 91.73, 82.98, 76.13, 68.42, 60.3, 26.12 (SiC(*C*H_3)$_3$), 25.87 (SiC(*C*H_3)$_3$), 25.82 (SiC(*C*H_3)$_3$), 18.61 (Si*C*(CH_3)$_3$), 18.06 (Si*C*(CH_3)$_3$), -4.03 (Si(*C*H_3)$_2$), -4.16 (Si(*C*H_3)$_2$), -5.11 (Si(*C*H_3)$_2$), -5.23 (Si(*C*H_3)$_2$), -5.57 (Si(*C*H_3)$_2$); MALDI-TOF (m/z) calcd for $C_{28}H_{56}N_2O_5Si_3$ = 637.33, found 638.31 (M+H^+)

^{1}H NMR of **3**:

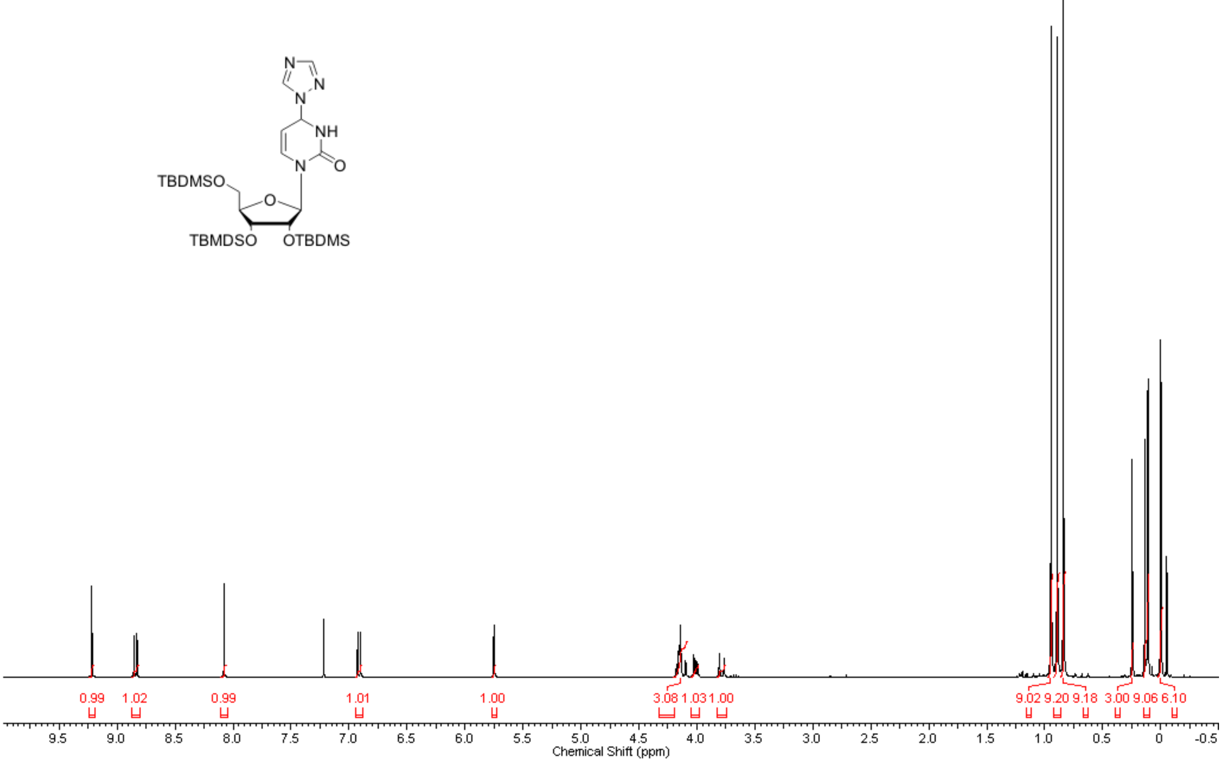

^{13}C NMR of **3**:

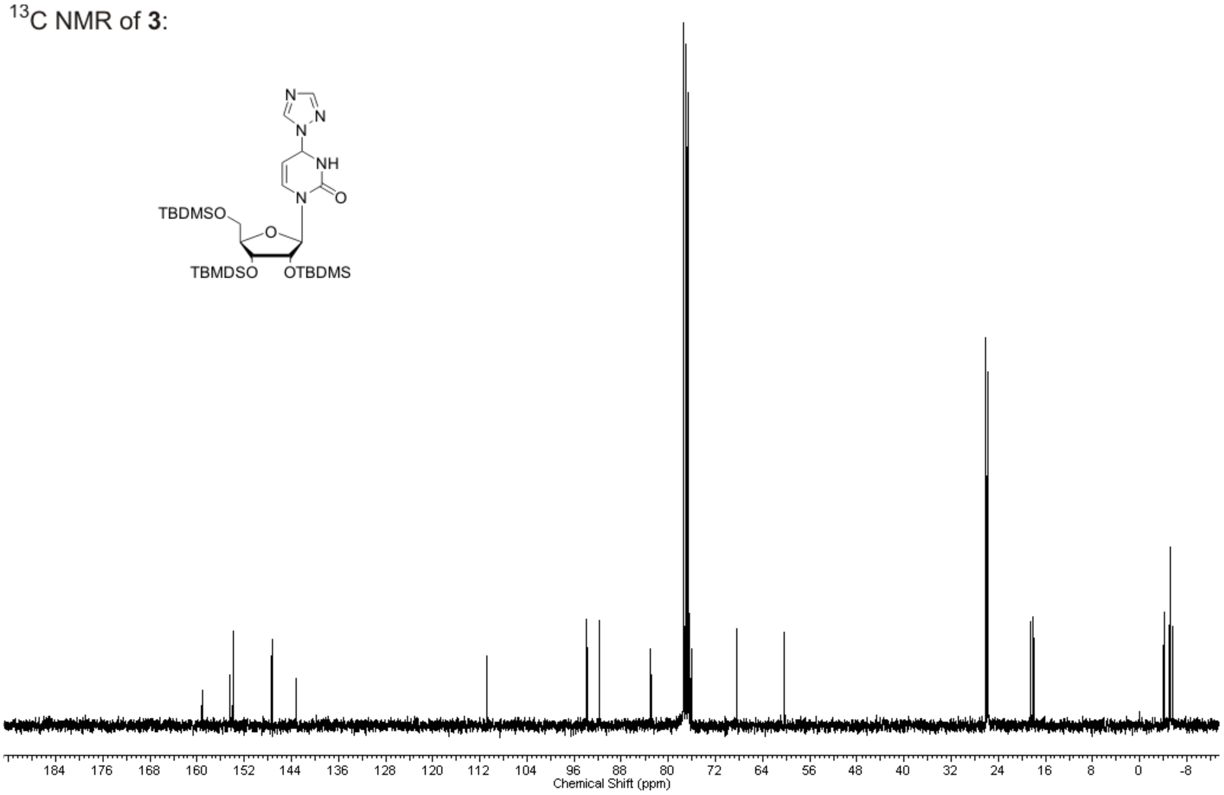

Synthesis of *N*-(*tert*-butoxycarbonyl)-3,6-dioxaoctan-1,8-diamine (4)

To a solution of 2,2'-(ethylenedioxy)diethylamine (17.78 g, 120 mmol) in dioxane (80 mL) a solution of di-*tert*-butyldicarbonate (4.36 g, 20 mmol) in dioxane (30 mL) was added over a period of 5 h. The reaction was stirred for 3 h at rt. The solvents were evaporated under reduced pressure. The residue was dissolved in H_2O (20 mL) and extracted with DCM (45 mL). The organic layer was washed with saturated aq. NaCl and dried over Na_2SO_4. The solution was concentrated under diminished pressure and the residue chromatographed on a silica gel column with ethyl acetate-hexane (1:2 → 2:1, v/v) → DCM-methanol-NH_3 (80:15:1.5, v/v/v) to give **4** (3.41 g, 68%) as a colorless syrup. ^{1}H NMR (300 MHz, $CDCl_3$) δ (ppm) 5.21 (br, 1 H, N*H*), 3.62-3.46 (m, 8 H, CH_2-O), 3.32 (q, *J* = 5.2 Hz, 2 H, CH_2-NH), 2.86-2.92 m, 2 H, CH_2-NH_2), 1.96 (br, 2 H, NH_2), 1.45 (s, 9 H, C(CH_3)$_3$); ^{13}C NMR (300 MHz, $CDCl_3$) δ (ppm) 155.98 (Boc-*C*O), 79.33 (Boc-*C*(CH_3)$_3$), 73.17 (*C*H_2-O), 70.15 (*C*H_2-O), 41.57 (*C*H_2-NH_2), 40.29 (*C*H_2-NH), 28.36 (Boc-C(*C*H_3)$_3$); MALDI-TOF (m/z) calcd for $C_{11}H_{24}N_2O_4$ = 248.17, found 248.96 ($M+H^+$)

^{1}H NMR of **4**:

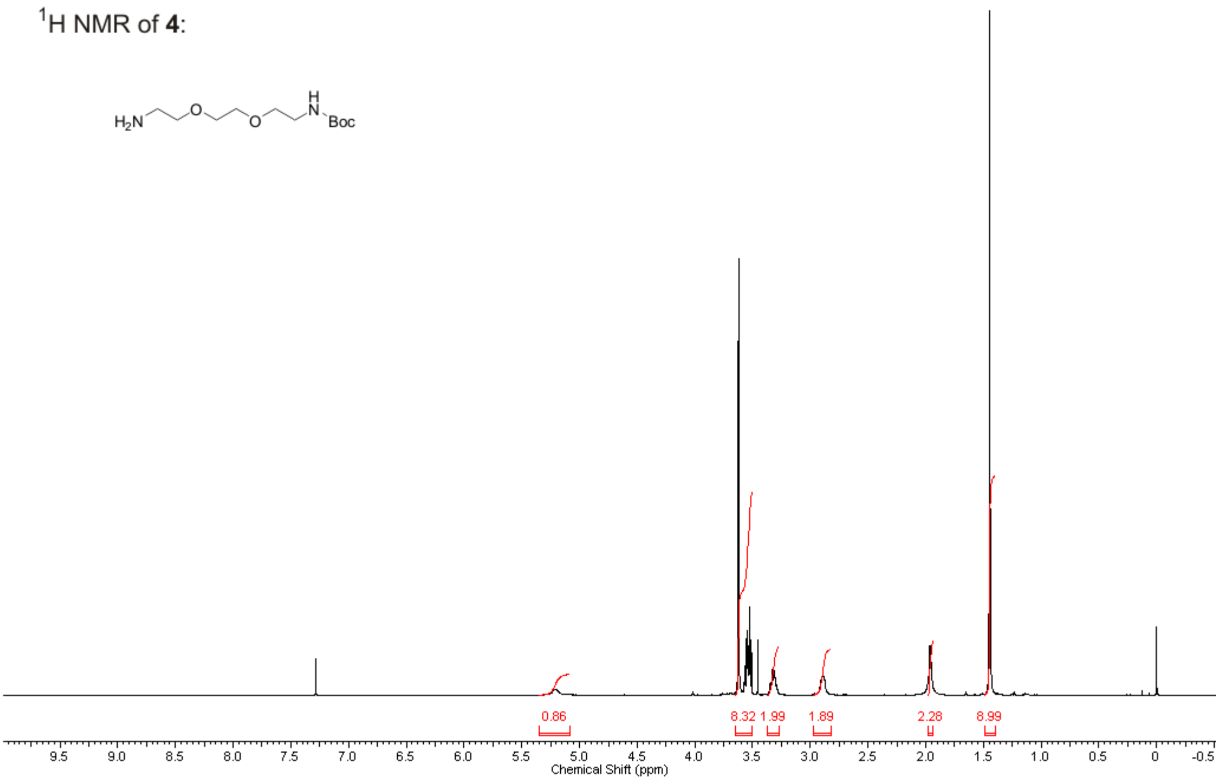

^{13}C NMR of **4**:

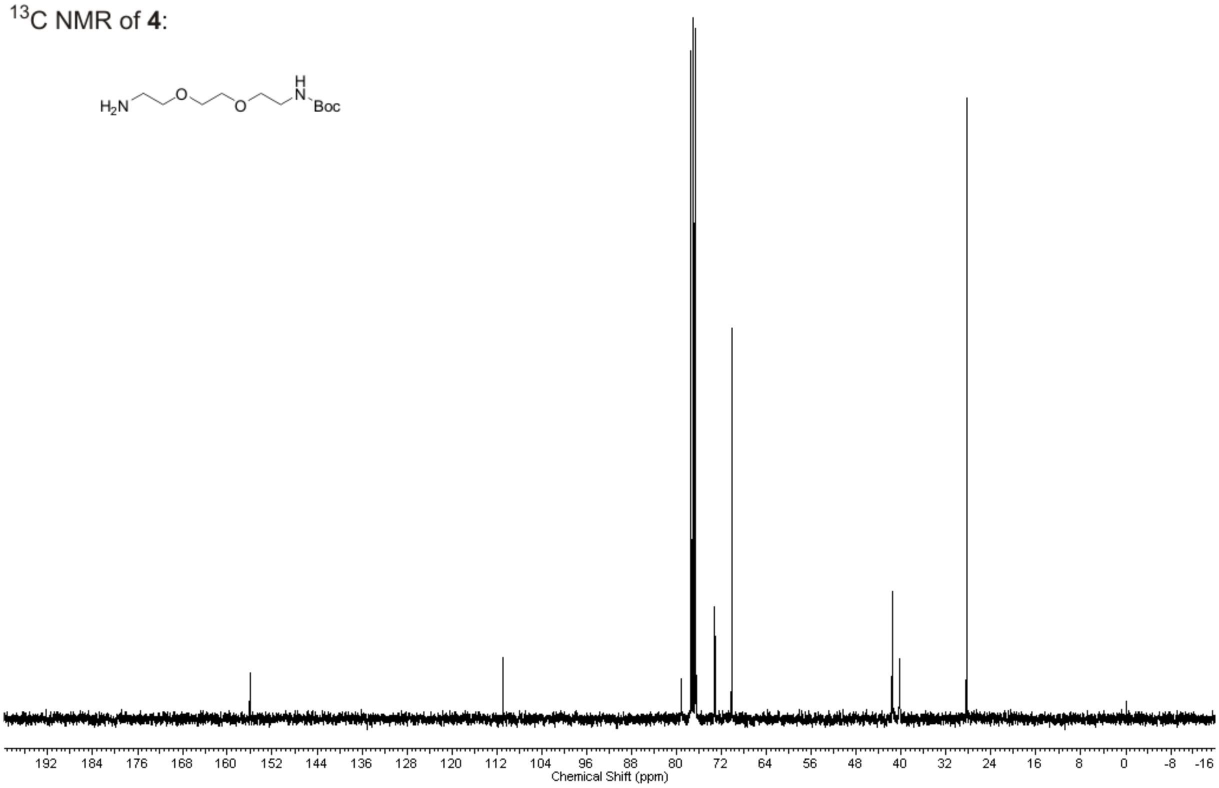

Synthesis of 2',3',5'-tris-*O*-(*tert*-butyldimethylsilyl)-1-[4-(*N*'-(*tert*-butoxycarbonyl)-3,6-dioxaoctane-1,8-diamine)pyrimidine-2(1*H*)-onyl]-*β*-D-riboside (9)

To a solution of compound **4** (0.64 g, 2.6 mmol) in dry acetonitrile (50 mL) the nucleoside **3** (1.27 g, 2 mmol) was added. The reaction mixture was stirred at rt overnight. The solvents were evaporated under reduced pressure. The residue was dissolved in DCM (50 mL), and the organic layer was washed with saturated aq. $NaHCO_3$ (30 mL), brine and dried over Na_2SO_4. After filtration, the solvents were evaporated and the crude product was chromatographed on a silica gel column with ethyl acetate-hexane (1:2, v/v) → DCM-methanol (95:5, v/v) to give **9** (1.38 g, 85%). ^{1}H NMR (300 MHz, $CDCl_3$) δ (ppm) 8.11 (br, 1 H, 6-C*H*), 5.72 (s, 1 H, 1'-C*H*), 5.66 (br, 1 H, N*H*), 5.55 (br, 1 H, 5-C*H*), 5.0 (br, 1 H, N*H*), 4.15-4.0 (m, 4 H, 2'-C*H*, 3'-C*H*, 4'-C*H*, 5'-C*H*), 3.73-3.79 (m, 1 H, 5''-C*H*), 3.67-3.54 (m, 10 H, CH_2-O + CH_2-NH), 3.25-3.35 (m, 2 H, CH_2NH), 1.44 (s, 9 H, $C(CH_3)_3$), 0.94 (s, 9 H, Si-$C(CH_3)_3$), 0.91 (s, 9 H, Si-$C(CH_3)_3$), 0.88 (s, 9 H, Si-$C(CH_3)_3$), 0.25 (s, 3 H, Si-$(CH_3)_2$), 0.13 (s, 6 H, Si-$(CH_3)_2$), 0.11 (s, 3 H, Si-$(CH_3)_2$), 0.04 (2 x s, 6 H, Si-$(CH_3)_2$); ^{13}C NMR (300 MHz, $CDCl_3$) δ (ppm) 163.75 (4-*C*N), 156.1 (2-*C*O), 156.01 (Boc-*C*O), 140.35 (6-*C*H), 93.98 (5-*C*H), 90.68, 82.48, 79.43 (Boc-$C(CH_3)_3$), 76.16, 70.28 (CH_2-O), 68.98, 60.72, 40.98 (CH_2-NH), 40.31 (CH_2-NH), 28.39 (Boc-$C(CH_3)_3$), 26.06 Si$C(CH_3)_3$), 25.91 (Si$C(CH_3)_3$), 25.84 (Si$C(CH_3)_3$), 18.49 (Si$C(CH_3)_3$), 18.03 (Si$C(CH_3)_3$), -4.1 (Si$(CH_3)_2$), -4.17 (Si$(CH_3)_2$), -5.11 (Si$(CH_3)_2$), -5.21 (Si$(CH_3)_2$), -5.33 (Si$(CH_3)_2$), -5.6 (Si$(CH_3)_2$); MALDI-TOF (m/z) calcd for $C_{38}H_{76}N_4O_9Si_3$ = 816.49, found 817.75 (M+H^+)

^{1}H NMR of **9**:

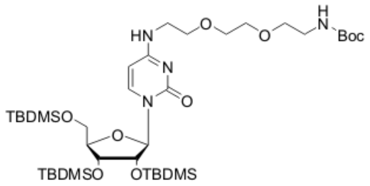

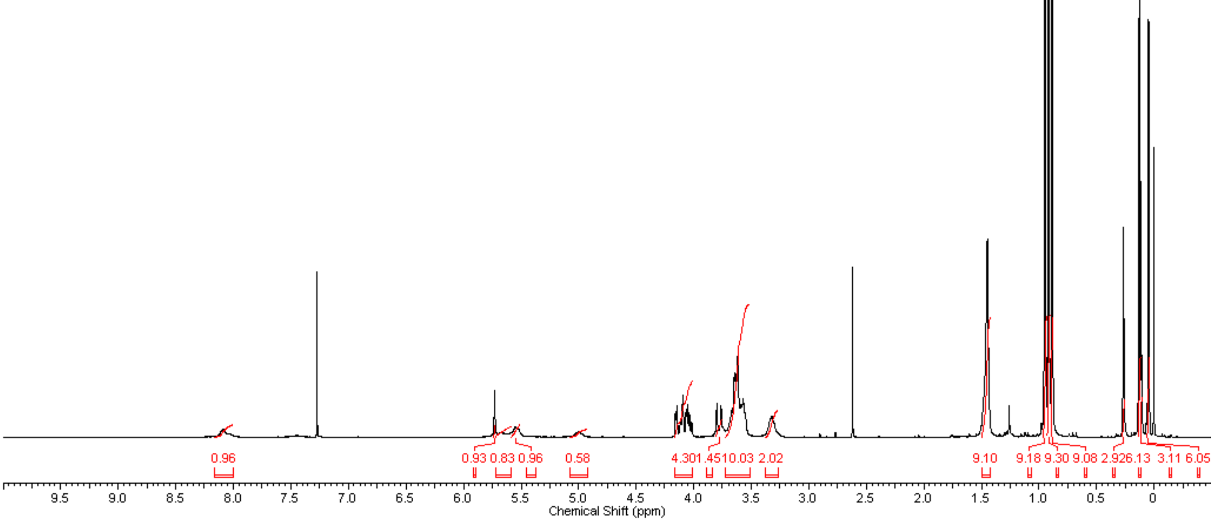

^{13}C NMR of **9**:

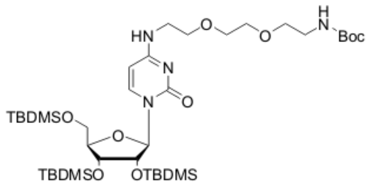

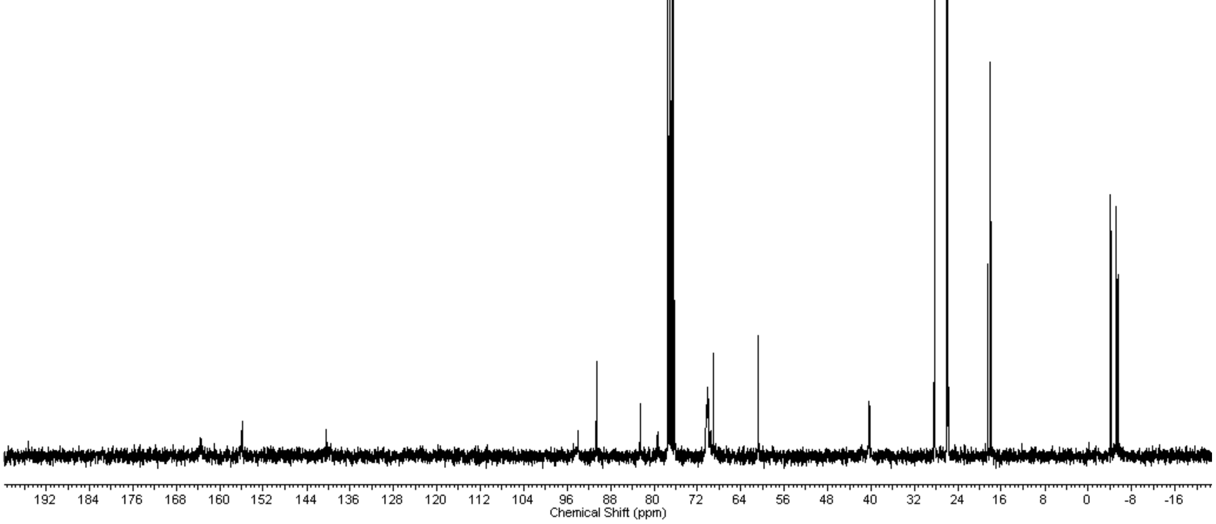

Synthesis of 2',3',5'-tris-*O*-(*tert*-butyldimethylsilyl)-1-[4-(3,6-dioxaoctane-1,8-diamine)pyrimidine-2(1*H*)-onyl]-β-D-riboside (10)

To a solution of **9** (680 mg, 0.83 mmol) in absolute DCM (50 mL) anhydrous $ZnBr_2$ (504 mg, 2.2 mmol) was added. The reaction mixture was stirred at rt for 1 d. The reaction was quenched with 1 M Na_2CO_3 (250 mL) and the aqueous layer was extracted three times with diethyl ether. The organic layer was dried over Na_2SO_4, filtered and concentrated under reduced pressure. The residue was chromatographed on a silica gel column with DCM-methanol (95:5, v/v) → DCM-methanol-NH_3 (94.5:4.5:1) to give compound **10** (490 mg, 82%) as a colorless foam. ^{1}H NMR (300 MHz, $CDCl_3$) δ (ppm) 8.01 (d, *J* = 7.4 Hz, 1 H, 6-C*H*), 6.58 (br, 1 H, N*H*), 5.75 (d, *J* = 1.7 Hz, 1 H, 1'-C*H*), 5.68 (d, *J* = 7.4 Hz, 1 H, 5-C*H*), 4.11-4.0 (m, 4 H, 2'-C*H*, 3'-C*H*, 4'-C*H*, 5'-C*H*), 3.75-3.79 (m, 1 H, 5'-C*H*), 3,73-3,61 (m, 10 H, CH_2-O + CH_2- NH), 2.97 (m, 2 H, CH_2NH_2), 2.74 (br, 2 H, NH_2), 0.94 (s, 9 H, Si-C(CH_3)$_3$), 0.90 (s, 9 H, Si-C(CH_3)$_3$), 0.88 (s, 9 H, Si-C(CH_3)$_3$), 0.21 (s, 3 H, Si-(CH_3)$_2$), 0.12 (s, 3 H, Si-(CH_3)$_2$), 0.11 (s, 3 H, Si-(CH_3)$_2$), 0.1 (s, 3 H, Si-(CH_3)$_2$), 0.06 (s, 3 H, Si-(CH_3)$_2$), 0.05 (s, 3 H, Si-(CH_3)$_2$); ^{13}C NMR (300 MHz, $CDCl_3$) δ (ppm) 163.8 (4-*C*N), 156.47 (2-*C*O), 139.95 (6-*C*H), 94.65 (5-*C*H), 90.39, 82.81, 76.18, 70.14 (*C*H_2-O), 70.02 (CH_2-O), 69.77 (*C*H_2-O), 68.36, 60.98, 40.37 (*C*H_2-NH), 26.06 (SiC(*C*H_3)$_3$), 25.89 (SiC(*C*H_3)$_3$), 25.9 (SiC(*C*H_3)$_3$), 18.5 (Si*C*(CH_3)$_3$), 18.04 (Si*C*(CH_3)$_3$), -4.11 (Si(CH_3)$_2$), -4.22 (Si(*C*H_3)$_2$), -5.05 (Si(*C*H_3)$_2$), -5.12 (Si(*C*H_3)$_2$), -5.34 (Si(*C*H_3)$_2$), -5.56 (Si(*C*H_3)$_2$); MALDI-TOF (m/z) calcd for $C_{33}H_{68}N_4O_7Si_3$ = 716.44, found 717.65 ($M+H^+$)

^{1}H NMR of **10**:

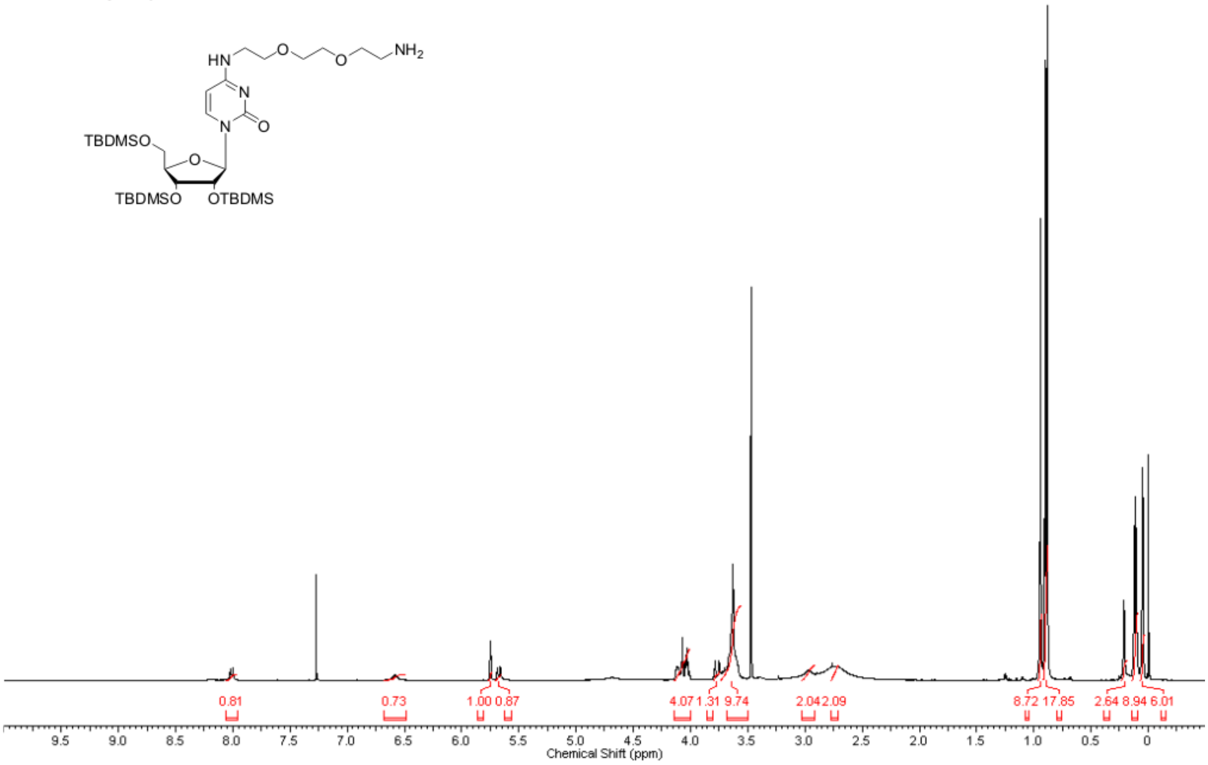

^{1}H NMR of **10**:

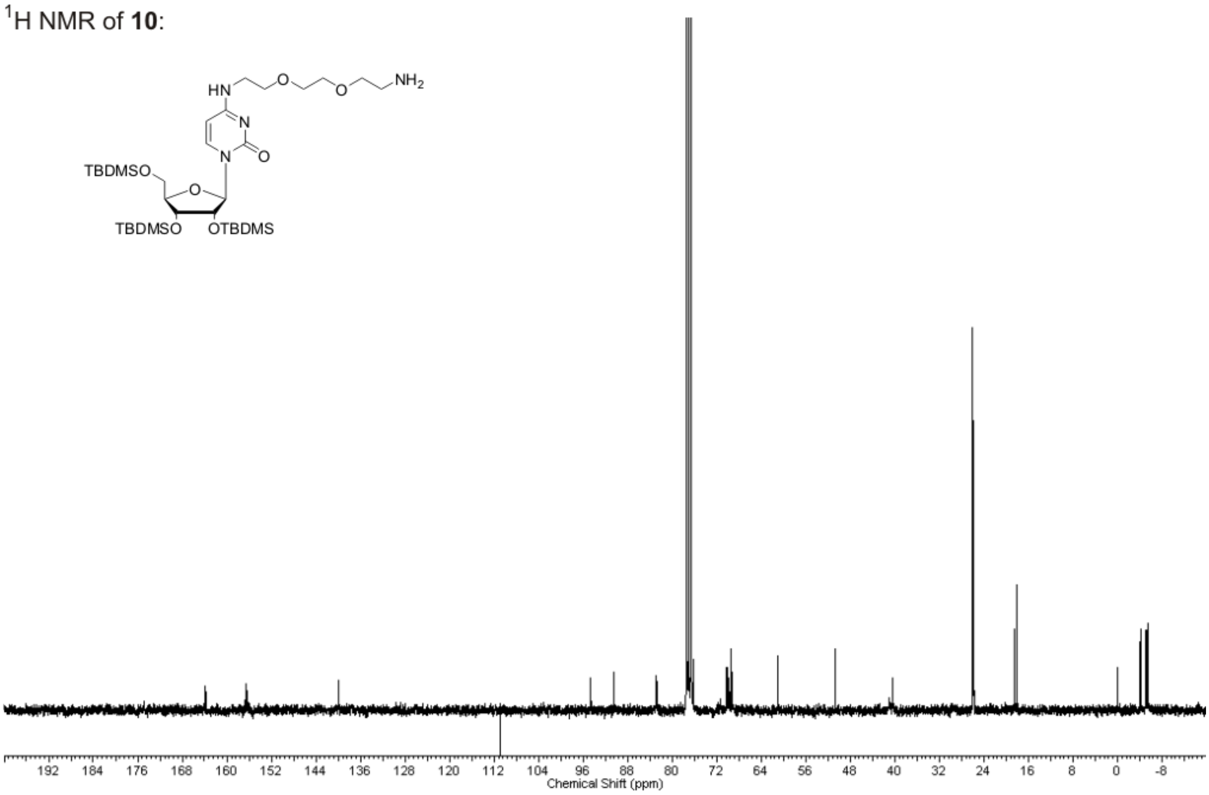

Synthesis of 2',3',5'-tris-*O*-(*tert*-butyldimethylsilyl)-1-[4-*N*'-biotinyl-3,6-dioxa-octane-1,8-diaminepyrimidine-2(1*H*)-onyl]-*β*-D-riboside (11)

To a chilled solution of compound **10** (500 mg, 0.7 mmol) in anhydrous DMF (20 mL) biotin (188 mg, 0.77 mmol) and *N*-(3-dimethylaminopropyl)-*N′*-ethylcarbodiimide hydrochloride (147 mg, 0.77 mmol) were added. The reaction mixture was stirred at rt overnight. After evaporation of the solvents, the residue was dissolved in DCM, and the organic layer was extracted washed with saturated aq. $NaHCO_3$ and brine. The crude product was dried over Na_2SO_4, filtered and chromatographed on a silica gel column with DCM-methanol (95:5 → 9:1) to yield **11** (430 mg, 65%). ^{1}H NMR (300 MHz, $CDCl_3$) δ (ppm) 8.04 (d, *J* = 7.4 Hz, 1 H, 6-C*H*), 6.68 (br, 1 H, N*H*), 6.48 (br, 1 H, N*H*), 6.24 (br, 1 H, N*H*), 5.74 (s, 1 H), 5.63 (d, *J* = 7.5 Hz, 1 H, 5-C*H*), 5.55 (s, 1 H, N*H*), 4.49-4.53 (m, 1 H, Biotin-C*H*NH), 4.29-4.33 (m, 1 H, Biotin-C*H*NH), 4.14-4.01 and 3.79-4.76 (m, 5 H, 2'-C*H*, 3'-C*H*, 4'-C*H*, 5'-C*H*), 3.65-3.57 (m, 10 H, CH_2-O + C*H*-NH), 3.48-3.39 (m, 2 H, CH_2NH), 3.18-3.12 (m, 1 H, C*H*S), 2.9 and 2.74 (m, 2 H, CH_2S), 2.22 (t, *J* = 7,1 Hz, 2 H, CH_2CONH), 1.71-1.64 (m, 4 H, CH_2CH$_2$CH_2), 1.49-1.42 (m, 2 H, CH$_2$CH_2CH$_2$), 0.95 (s, 9 H, Si-C(CH_3)$_3$), 0.91 (s, 9 H, Si-C(CH_3)$_3$), 0.89 (s, 9 H, Si-C(CH_3)$_3$), 0.24 (s, 3 H, Si-(CH_3)$_2$), 0.13 (s, 3 H, Si-(CH_3)$_2$), 0.11 (s, 6 H, Si-(CH_3)$_2$), 0.05 (s, 6 H, Si-(CH_3)$_2$);^{13}C NMR (300 MHz, $CDCl_3$) δ (ppm) 173.36 (CH$_2$*C*ONH), 163.96 and 163.78 (4- *C*N) and NH*C*ONH), 156.15 (2-*C*O), 140.19 (6-*C*H), 94.28 (5-*C*H), 90.64, 82.68, 76.15, 70.14 (*C*H$_2$-O), 69.9 (*C*H$_2$-O), 69.75 (*C*H$_2$-O), 69.22, 61.74 (Biotin-*C*HN) 60.91, 60.19 (Biotin-*C*HN), 55.45 (*C*S), 40.46 (*C*H$_2$S), 39.12 (Biotin-*C*H$_2$), 35.96 (Biotin-*C*H$_2$), 28.0 (Biotin-*C*H$_2$), 26.1 (SiC(*C*H$_3$)$_3$), 25.92 (SiC(*C*H$_3$)$_3$), 25.86 (SiC(*C*H$_3$)$_3$), 25.48 (Biotin-*C*H$_2$), 18.52 (Si*C*(CH$_3$)$_3$), 18.05 (Si*C*(CH$_3$)$_3$), -4.09 (Si(*C*H$_3$)$_2$), -4.16 (Si(*C*H$_3$)$_2$), -5.05 (Si(*C*H$_3$)$_2$), -5.14 (Si(*C*H$_3$)$_2$), -5.3 (Si(*C*H$_3$)$_2$), -5.53 (Si(*C*H$_3$)$_2$); MALDI-TOF (m/z) calcd for $C_{43}H_{82}N_6O_9SSi_3$ = 942.52, found 943.81 ($M+H^+$)

^{1}H NMR of **11**:

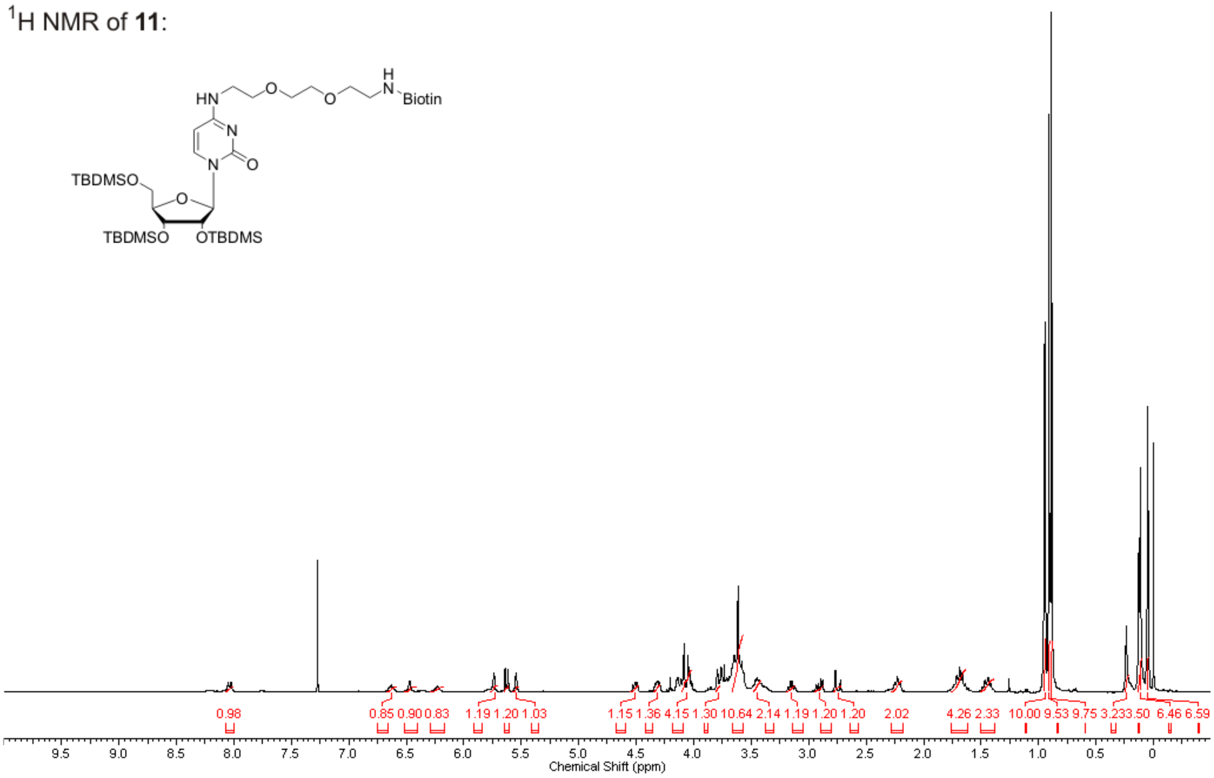

^{13}C NMR of **11**:

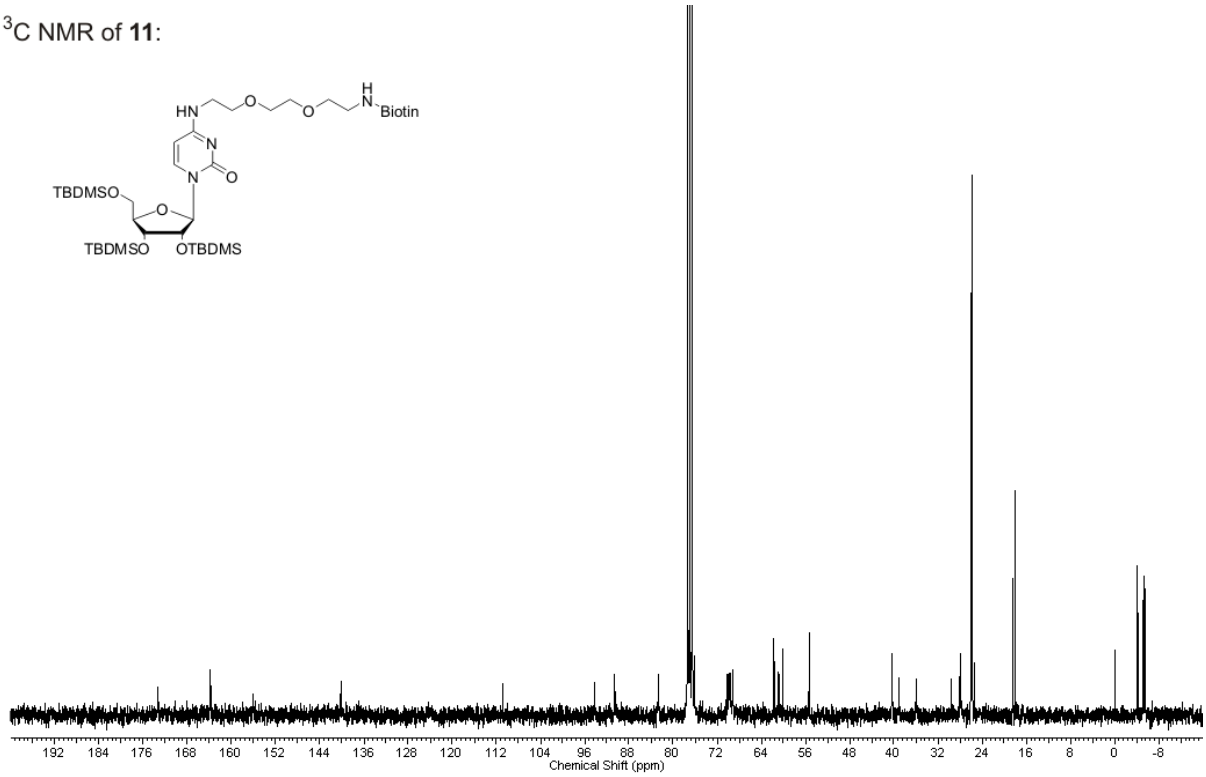

Synthesis of 2',3'-bis-*O*-(*tert*-butyldimethylsilyl)-1-[4-*N*'-biotinyl-3,6-dioxa-octane-1,8-diaminepyrimidine-2(1*H*)-onyl]-β -D-riboside (12)

A mixture of TFA-H_2O-THF (10 mL, 1:1:4, v/v/v) was cooled to 0 °C and added to compound **11** (220 mg, 230 µmol). The solution was stirred at 0 °C for 5 h and then quenched with saturated aq $NaHCO_3$. After addition of ethyl acetate (100 mL) the organic layer was washed with H_2O (10 mL), brine (10 mL) and dried over Na_2SO_4. The solvents were removed under reduced pressure and the residue chromatographed on a silica gel column with DCM-methanol (9:1) to yield **12** (179 mg, 94%) as a colorless foam. ^{1}H NMR (300 MHz, $CDCl_3$) δ (ppm) 7.45 (d, *J* = 7.4 Hz, 1 H, 6-C*H*), 6.89 (br, 1 H, N*H*), 6.77 (br, 1 H, N*H*), 6.73 (br, 1 H, N*H*), 5.82 (s, 1 H, 1'-C*H*), 5.78 (d, *J* = 7.4 Hz, 1 H, 5-C*H*), 5.31 (d, *J* = 5.2 Hz, 1 H O*H*), 4.75 (t, *J* = 5.0 Hz, 1 H, N*H*), 4.47 (m, 1 H, Biotin-C*H*NH), 4.27 (m, 1 H, Biotin-C*H*NH), 4.17 (m, 1 H), 4.04 (m, 1 H), 3.88 (m, 1 H), 3.63-3.52 (m, 10 H, CH_2-O + C*H*-NH), 3.40 (m, 2 H, CH_2NH), 3.10 (m, 1 H, C*H*S), 2.86-2.71 (m, 2 H, CH_2S), 2.20 (t, *J* = 7.3 Hz, 2 H, CH_2CONH), 1.64 (m, 4 H, $CH_2CH_2CH_2$), 1.39 (m, 2 H, $CH_2CH_2CH_2$), 0.87 (s, 9 H, Si-$C(CH_3)_3$), 0.83 (s, 9 H, Si-$C(CH_3)_3$), 0.08 (2 x s, 6 H, 2 x Si-$(CH_3)_2$), 0.05 (s, 3 H, Si-$CH_3)_2$), 0.01 (s, 3 H, Si-$(CH_3)_2$); ^{13}C NMR (300 MHz, $CDCl_3$) δ (ppm) 173.59 (CH_2*C*ONH), 164.40 and 163.90 (4- *C*N) and NH*C*ONH), 156.32 (2-*C*O), 143.37 (6-*C*H), 95.96 (5-*C*H), 95.40, 86.57, 72.86, 72.09, 70.08 (*C*H_2-O), 69.83 (*C*H_2-O), 69.40 (*C*H_2-O), 66.29, 61.77 (Biotin-*C*HN), 60.26 (Biotin-*C*HN), 55.55 (*C*S), 40.41 (*C*H_2S), 39.16 (Biotin-*C*H_2), 35.89 (Biotin-*C*H_2), 28.04 (Biotin-*C*H_2), 25.85 (SiC$(CH_3)_3$), 25.43 (Biotin-*C*H_2), 18.05 (Si*C*$(CH_3)_3$), 17.92 (Si*C*$(CH_3)_3$), -4.47 (Si$(CH_3)_2$), -4.69 (Si$(CH_3)_2$), -4.84 (Si$(CH_3)_2$); MALDI-TOF (m/z) calcd for $C_{37}H_{68}N_6O_9SSi_2$ = 828.43 found 851.90 ($M+Na^+$)

^{1}H NMR of **12**:

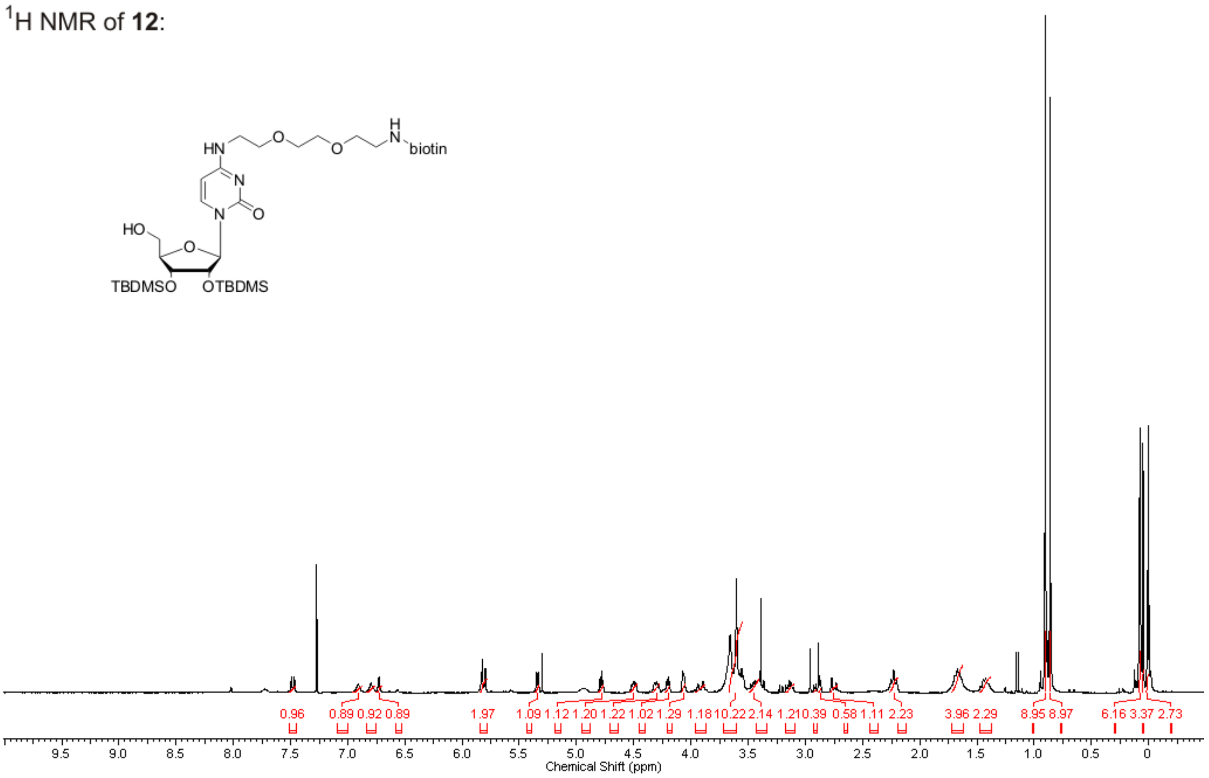

^{13}C NMR of **12**:

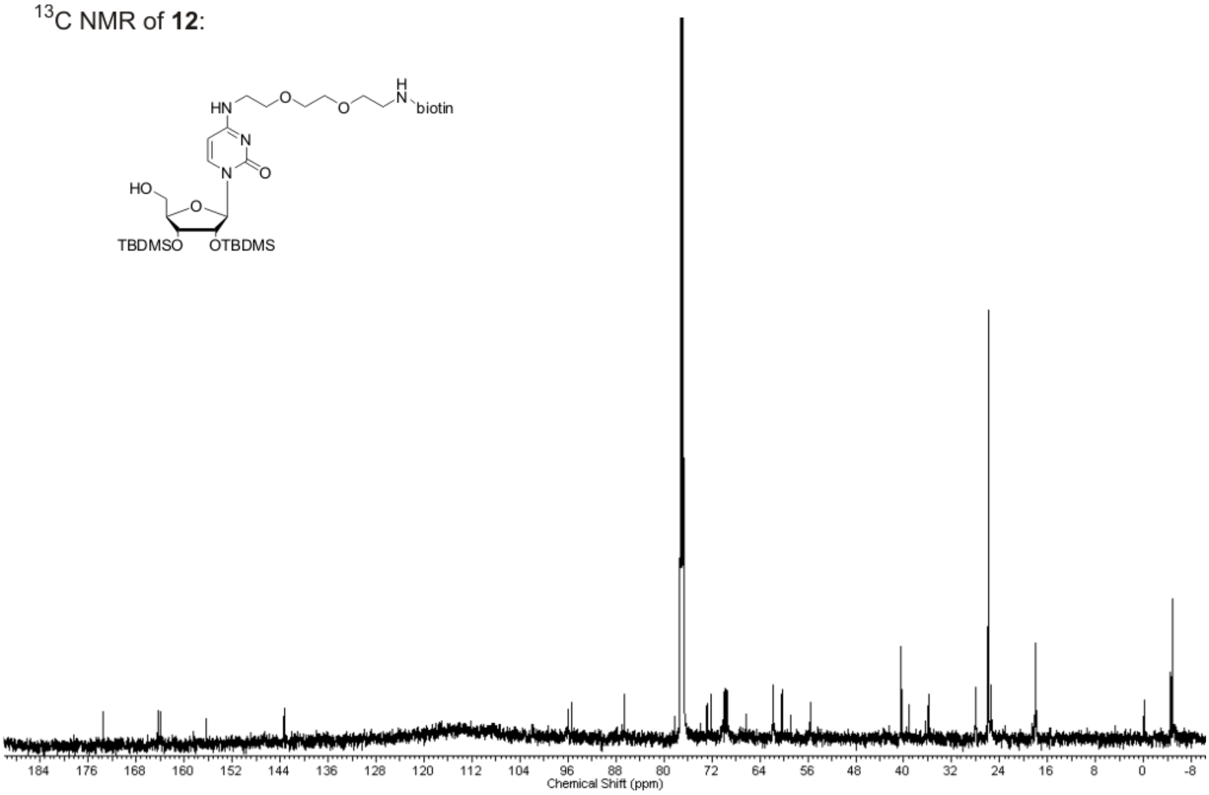

Synthesis of*N*-(*tert*-butoxycarbonyl)-17-amino-3,6,9,12,15-pentaoxaheptadecane-1-ol (14)

To a solution of linker **13** (0.65 g, 2.3 mmol) in ethyl acetate (15 mL) di-*tert*-butyl dicarbonate (0.56 g, 2.6 mmol) was added. The reaction mixture was stirred at 60 °C for 90 min. The solution was cooled to rt and diluted with ethyl acetate (30 mL). The organic layer was washed two times with brine (20 mL each), dried over Na_2SO_4, filtered and concentrated under reduced pressure. The crude product was chromatographed on a silica gel column with DCM-methanol (9:1) to give compound **14** (797 mg, 98%) as a clear syrup.^{1}H NMR (300 MHz, $CDCl_3$) δ (ppm) 3.74-3.52 (m, 22 H, CH_2CH_2-O), 3.31 (br, 2 H, CH_2-NH), 2.76 (br, 1 H, O*H*), 1.44 (s, 9 H, $C(CH_3)_3$); ^{13}C NMR (300 MHz, $CDCl_3$) δ (ppm) 155.98 (Boc-*C*O), 79.33 ($C(CH_3)_3$), 76.58 (CH_2-O), 72.48 (CH_2-O), 70.54 (CH_2-O), 70.30 (CH_2-O), 70.19 (CH_2-O), 61.67 (CH_2-OH), 40.34 (CH_2-NH), 28,39 ($C(CH_3)_3$); MALDI-TOF (m/z) calcd for $C_{17}H_{35}NO_8$ = 381.24 found 404.19 ($M+Na^+$)

^{1}H NMR of **14**:

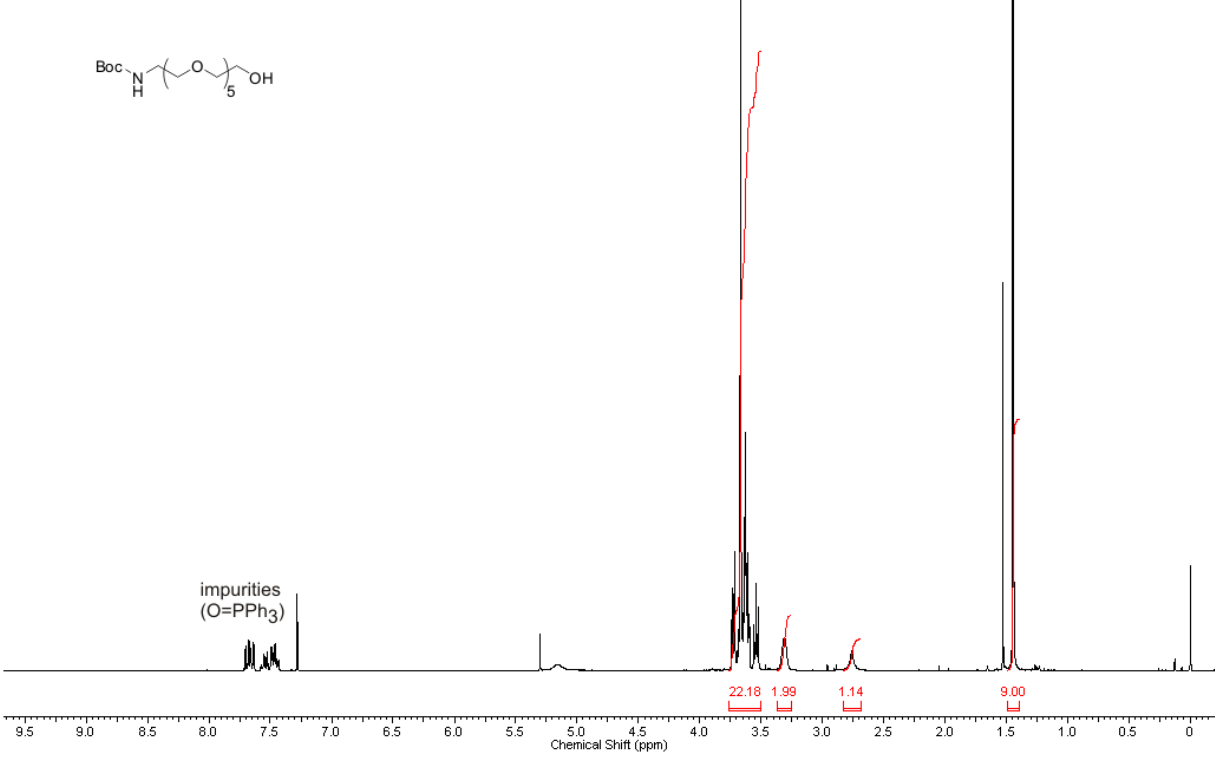

^{1}H NMR of **14**:

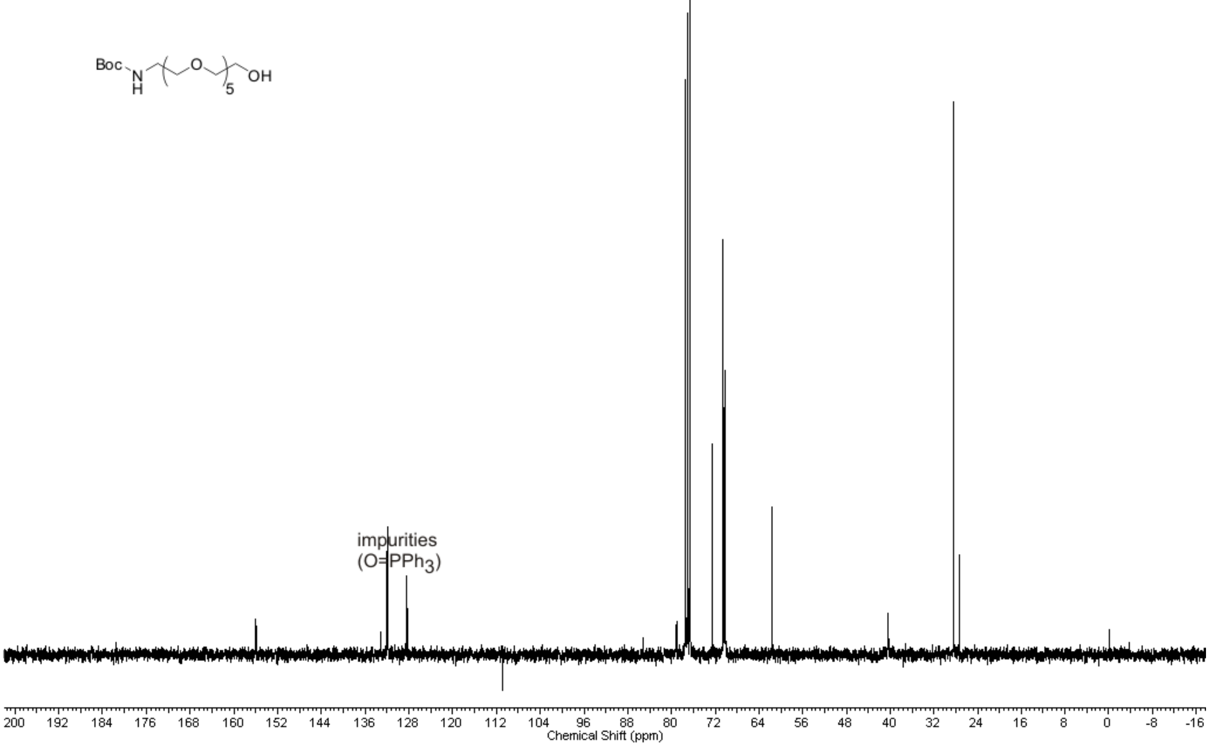

Synthesis of *N*-(*tert*-butoxycarbonyl)hexaethyleneglycolmonoaminephosphor-amidite (2)

Compound **14** (400 mg, 1 mmol) was coevaporated with anhydrous DCM three times and subsequently dissolved in DCM (15 mL). Freshly distilled (over CaH_2) DIPEA (650 µL, 5.3 mmol) was added. After dropwise addition of 2-cyanoethyl *N*,*N*-diisopropylchlorophosphoramidite (300 µL, 1.3 mmol), the solution was stirred at rt for 90 min. The reaction was quenched with anhydrous methanol (1 mL) and diluted with ethyl acetate (15 mL) and TEA (1 mL). The organic layer was washed with saturated aq. $NaHCO_3$ and brine (10 mL each) and dried over Na_2SO_4. After filtration of the drying agent, the solvents were removed under diminished pressure. The crude phosphoramidite **2** (468 mg, 80%) was coevaporated three times with anhydrous DCM and used in the next reaction without further purification. ^{31}P NMR (300 MHz, $CDCl_3$), two rotamers, δ (ppm) 149.42, 148.49

^{31}P NMR of **2**:

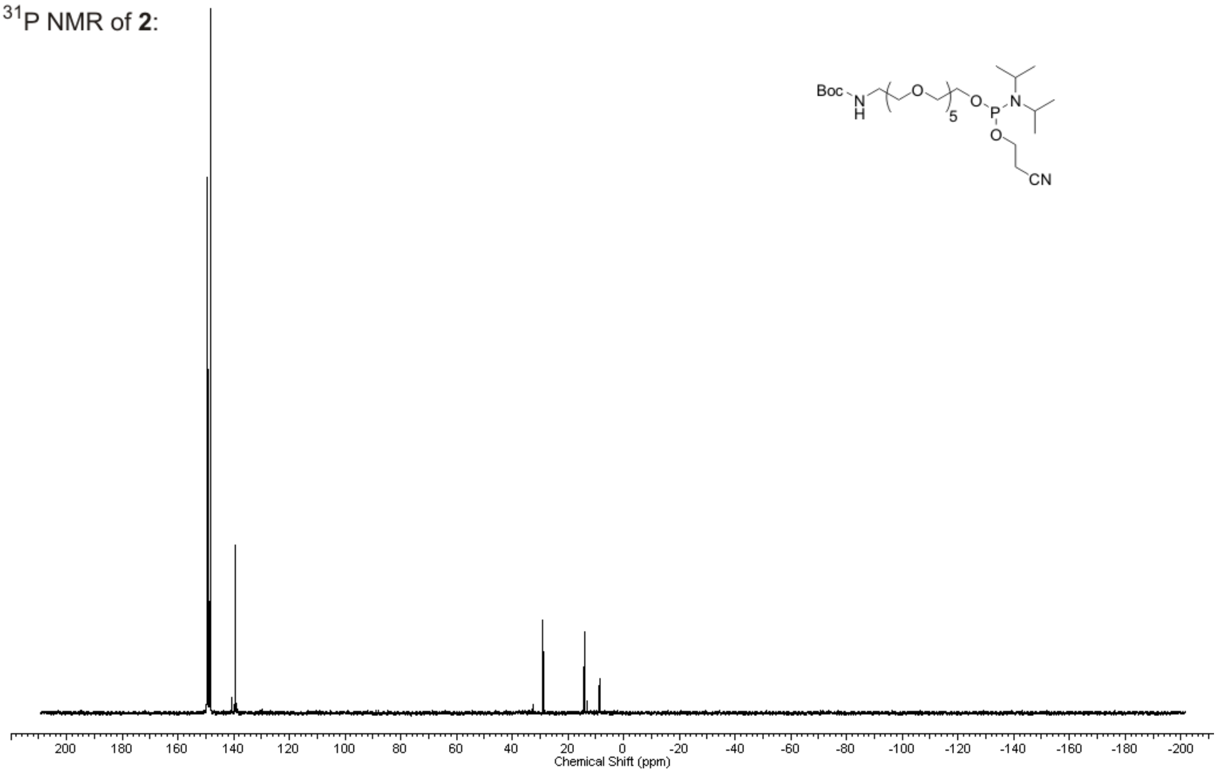
Boc
N
H
O
5
O
P
N
O
CN
200
180
160
140
120
100
80
60
40
20
0
-20
-40
-60
-80
-100
-120
-140
-160
-180
-200
Chemical Shift (ppm)

Synthesis of *N*^{4}-(3,6-dioxa-8-*N*'-biotinyl)-2',3'-bis-*O*-(*tert*-butyldimethylsilyl)-5'-*O*-β-cyanoethylphosphoryl-[(*N*-*tert*-butoxycarbonyl)-17-amino-3,6,9,12,15-pentaoxaheptadecyl]cytidine (16)

All compounds were coevaporated three times with anhydrous DCM before use. The cytidine derivative **12** (130 mg, 157 µmol) was dissolved in absolute THF (1 mL). To the solution the phosphoramidite **2** (100 mg, 173 µmol, dissolved in 1 mL THF) and 5-benzylmercaptotetrazol (151 mg, 785 µmol, dissolved in 1 mL THF) were added. The reaction mixture was stirred at rt for 90 min and subsequently quenched with 0.2 M iodine in THF/pyridine/H_2O (2:1:1, v/v/v). After 10 min of stirring at rt, aq. 5% $NaHSO_3$ was added until disappearance of the brownish color of iodine. The solution was diluted with DCM (10 mL), and the organic layer was washed with saturated aq. $NaHCO_3$ and brine. After drying over Na_2SO_4, the solvents were evaporated under reduced pressure. The crude product was purified on a reversed phase column to give **15** (58 mg, 28%) as a colorless foam. Column: VP Nucleodur 250 C18 (Macherey-Nagel); flow rate 4 ml/min; eluent (A) 5% aq. acetonitrile, eluent (B) 70% aq. acetonitrile; gradient 0%→70% (B) in 5 CV, 70%→100% (B) in 3 CV, 100% (B) 3 CV.

^{1}H NMR (300 MHz, $CDCl_3$), two diastereomers δ (ppm) 7.68 (2 x d, *J* = 7.6 Hz, 1 H, 6-C*H*), 6.68 (br, 1 H, N*H*), 6.27 (br, 1 H, N*H*), 5.85 (d, *J* = 7.6 Hz, 1 H, 5-C*H*), 5.57 (br, 1 H, N*H*), 5.13 (br, 1 H,N*H*), 4.45-4.56 (m, 2 H), 4.35-4.18 (m, 7 H), 3.69 (m, 1 H), 3.74-3.50 (m, 30 H, CH_2-O), 3.47-3.43 (m, 2 H, CH_2-NH), 3.30-3.35 (m, 2 H, CH_2-NH-Boc), 3.19-3.15 (m, 1 H, C*H*-S), 2.90 and 2.75 (m, 2 H, CH_2S), 2.85-2.81 (m, 2 H, CH_2-CN), 2.26-2.17 (m, 2 H, CH_2-CONH), 1.75-1.65 (m, 4 H, CH_2CH_2CH_2), 1.44 (m, 11 H, OC(CH_3)$_3$ and CH_2CH_2CH_2), 0.92 (s, 9 H, Si-C(CH_3)$_3$), 0.89 (s, 9 H, Si-C(CH_3)$_3$), 0.25 (2 x s, 3 H, Si-(CH_3)$_2$), 0.13 (2 x s, 3 H, Si-(CH_3)$_2$), 0.06 (s, 6 H, Si-

($C\mathit{H}_3)_2$); ^{31}P NMR (300 MHz, $CDCl_3$), two diastereomers δ (ppm) -1.65, -1,68; MALDI-TOF (m/z) calcd for $C_{57}H_{105}N_8O_{19}PSSi_2$ = 1324.65 found 1348.41 (M+Na$^+$)

^{1}H NMR of **16**:

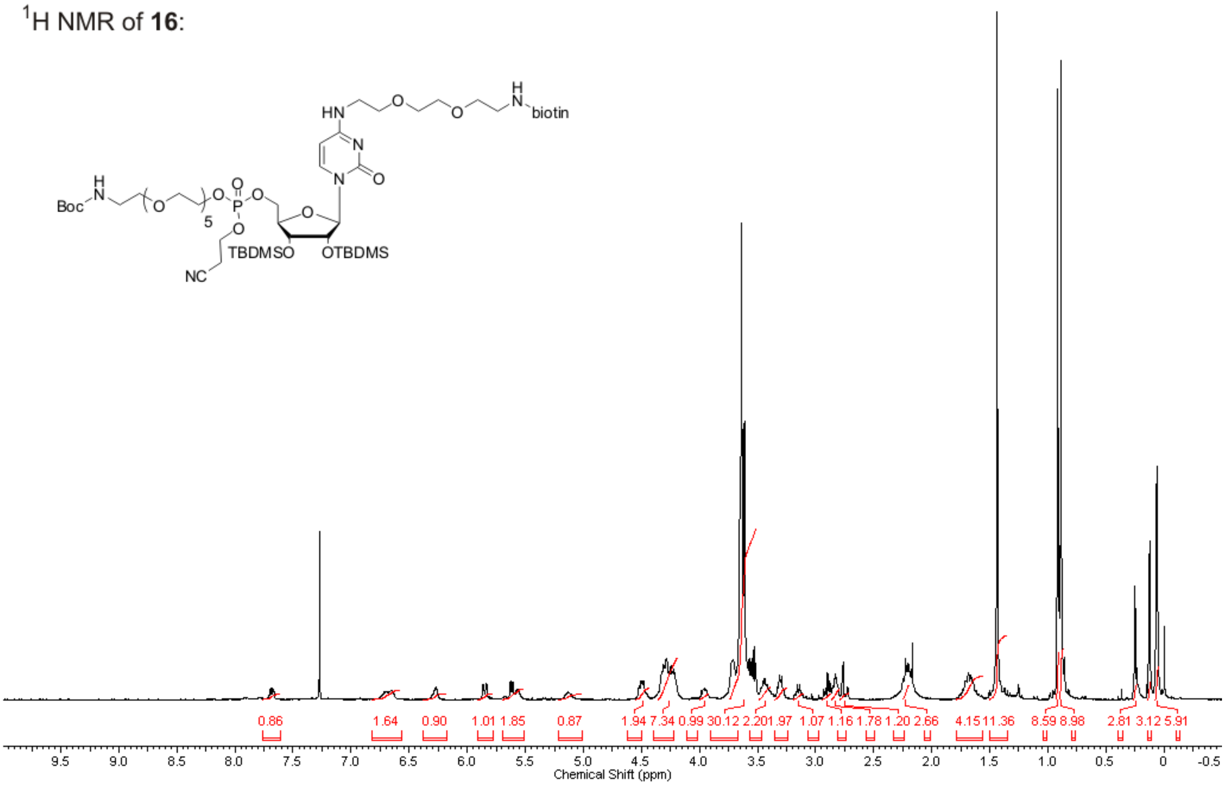

^{31}P NMR of **16**:

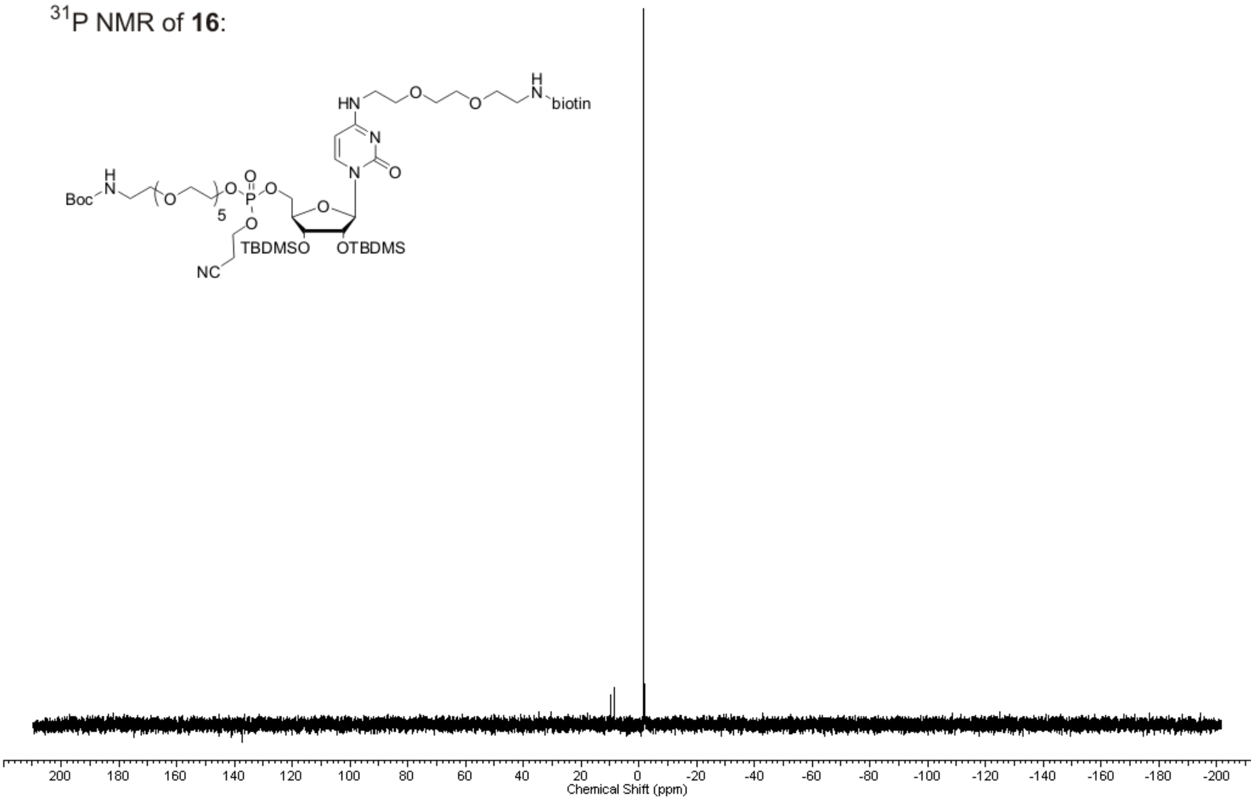

Synthesis of *N*^{4}-(3,6-dioxa-8-*N*'-biotinyl)-5'-*O*-phosphoryl-(17-amino-3,6,9,12,15-pentaoxaheptadecyl)cytidine (19)

The fully protected cytidine derivative **16** (20 mg, 15 µmol) was dissolved in 100 µl of a mixture of aq. NH_3 (30%)/methylamine (8 M) (1:1, v/v). After 30 min at rt, the solvents were evaporated and the residue was re-dissolved in 300 µl triethylamine trihydrofluoride and incubated for 2 h at 55 °C. The solvents were removed under reduced pressure, and the crude product was taken up in 100 µl neat TFA. After 2 min at rt, the volatile components were evaporated. Isolation of the deprotected cytidine was carried out via reversed phase chromatography to give the title compound **19** (11.7 mg; 83%) as a colorless foam. Column: VP Nucleodur 250 C18 (Macherey-Nagel); flow rate 4 ml/min; buffer (A): 0.1 M aq. triethylammonium acetate (pH 7.0); buffer (B) 0.1 M aq. triethylammonium acetate (pH 7.0), 70% acetonitrile; gradient 0%→40% (B) in 15 CV, 40%→100% (B) in 2 CV, 100% (B) in 1 CV.

^{1}H NMR (300 MHz, H_2O) δ (ppm) 7.77 (d, *J* = 7.6 Hz, 1 H, 6-C*H*), 6.01 (d, *J* = 7.6 Hz, 1 H, 5-C*H*), 5.93 (d, *J* = 3.8 Hz, 1 H, 1'-C*H*), 4.53 (dd, *J* = 7.9 Hz, *J* = 4.5 Hz, 1 H), 4.53 (dd, *J* = 7.9 Hz, *J* = 4.5 Hz, 1 H), 4.34 (dd, *J* = 7.9 Hz, *J* = 4.5 Hz, 1 H), 4.26-4.22 (m, 2 H), 4.17-4.14 (m, 2 H), 4.07-4.02 (m, 1 H), 4.01-3.91 (m, 2 H), 3.73-3,50 (m, 28 H, CH_2-O), 3.57-3.54 (m, 4 H), 3.32 (t, *J* = 5.3 Hz, 2 H, CH_2NH), 3.28-3.22 (m, 1 H, C*H*-S), 2.91 (dd, *J* = 13.2 Hz, *J* = 4.9 Hz, 1 H, CH_2-S), 2.70 (d, *J* = 13.2 Hz, 1 H, CH_2-S), 2.20 (t, *J* = 7.2 Hz, 2 H, CH_2-CONH), 1.76-1.48 (m, 4 H, CH_2CH_2CH_2), 1.38-1.26 (m, 2 H, CH_2C$H_2$$CH_2$), ^{13} C NMR (300 MHz, H_2O) δ (ppm) 181.19 (CH_2*C*NH), 165.07 and 163.98 (4-*C*N) and 163.98 (NH*C*ONH), 157.65 (2-*C*O), 139.50 (6-*C*H), 97.25 (5-*C*H), 88.99, 82.35, 82.22, 73.78, 69.96 (*C*H_2-O), 69.41 (*C*H_2-O), 69.33 (*C*H_2-O), 68.61 (*C*H_2-O), 68.33 (*C*H_2-O), 66.13 (*C*H_2-O), 64.5, 61.83 (biotin-*C*HN), 60.00

(biotin-*C*HN), 55.11 (*C*S), 39.90 (*C*H_2N), 39.46 (*C*H_2S), 38.90 (*C*H_2N), 38.62 (*C*H_2N), 35.23 (biotin-*C*H_2), 27.61 (biotin-*C*H_2), 27.46 (biotin-*C*H_2), 24.91 (biotin-*C*H_2); MALDI-TOF (m/z) calcd for $C_{37}H_{66}N_7O_{17}PS$ = 943.40 found 945.02 ($M+H^+$)

^{1}H NMR of **1**:

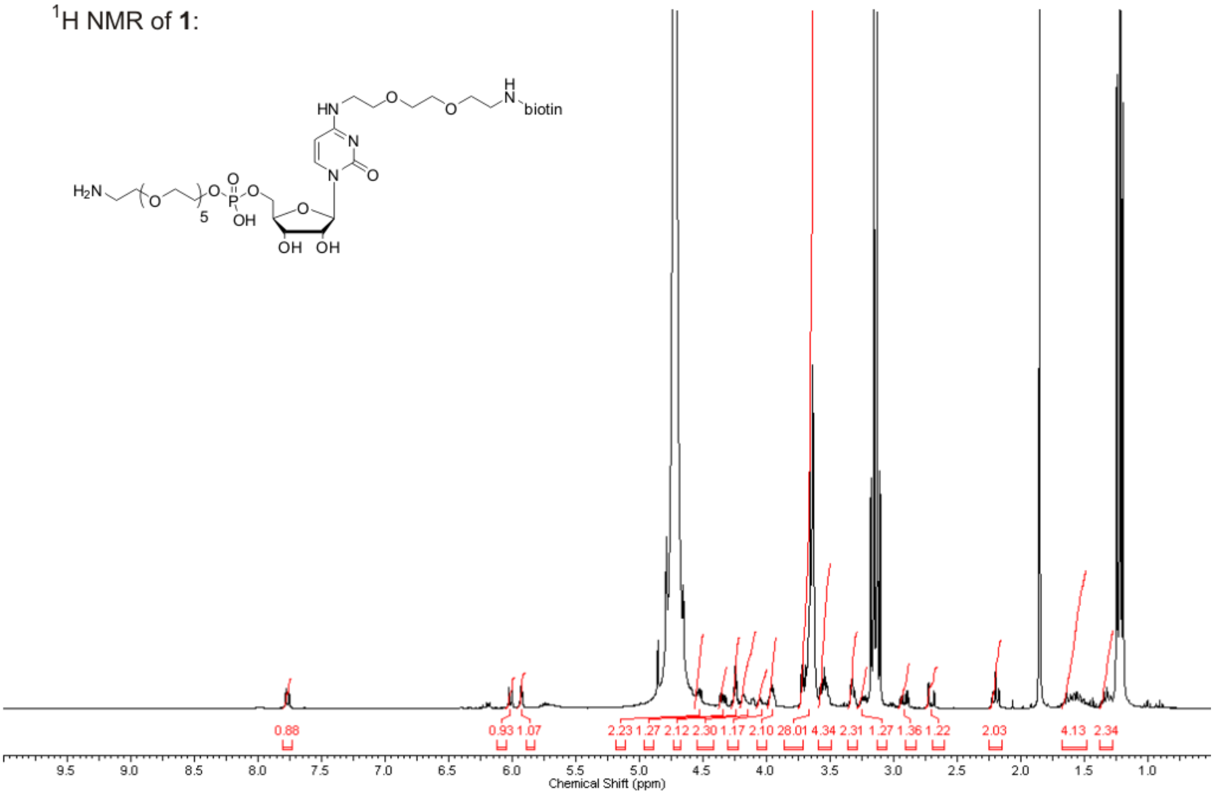

^{13}C NMR of **1**:

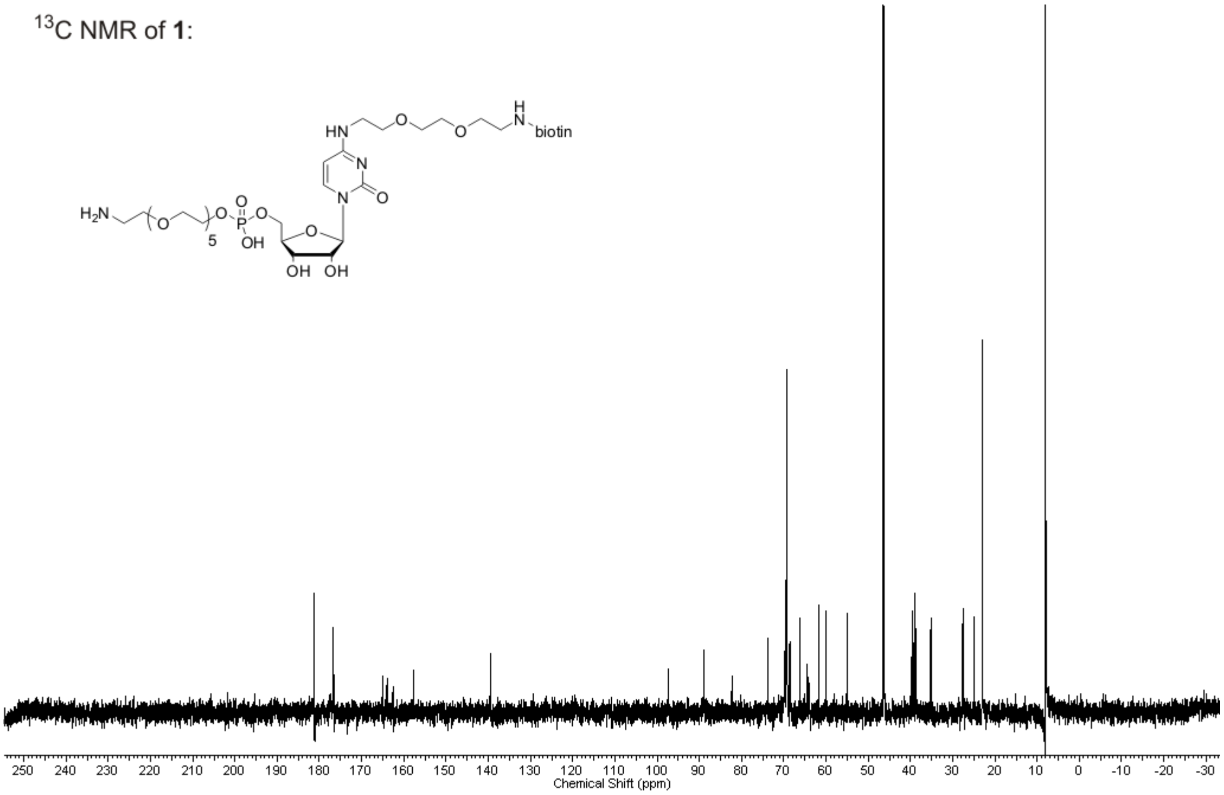

MALDI-Tof analysis of the coupling reaction between a short 20mer RNA substrate and cytidine derivative 1

The HPLC purified RNA-cytidine **1** adduct was dissolved in micropore water to a concentration of 200 pmol/µl. 2 µl of the solution were treated with cation exchange beads (Dowex 50 WX8, NH_4^+-form, 100–200 mesh, Serva Feinchemica). Therefore, 5 µl of a suspension of the beads in micropore water was transferred into a 250 µl eppendorf tube and dried by withdrawing the water with a 2.5 µl tip. To the beads the RNA sample was added and incubated for 10 minutes. The RNA was mixed with 1 µl of a matrix solution (60 mg THAP and 5.7 mg diammonium hydrogen citrate in a total volume of 2 ml) loaded onto the MALDI-plate and dried in the open air. The sample was measured using linear positive mode.

Calc. mass for the RNA-cytidine **1** adduct: 7339.9 g/mol; found 7339.04 $[M+1]^+$; 3668.515 $[M+2]^+/2$

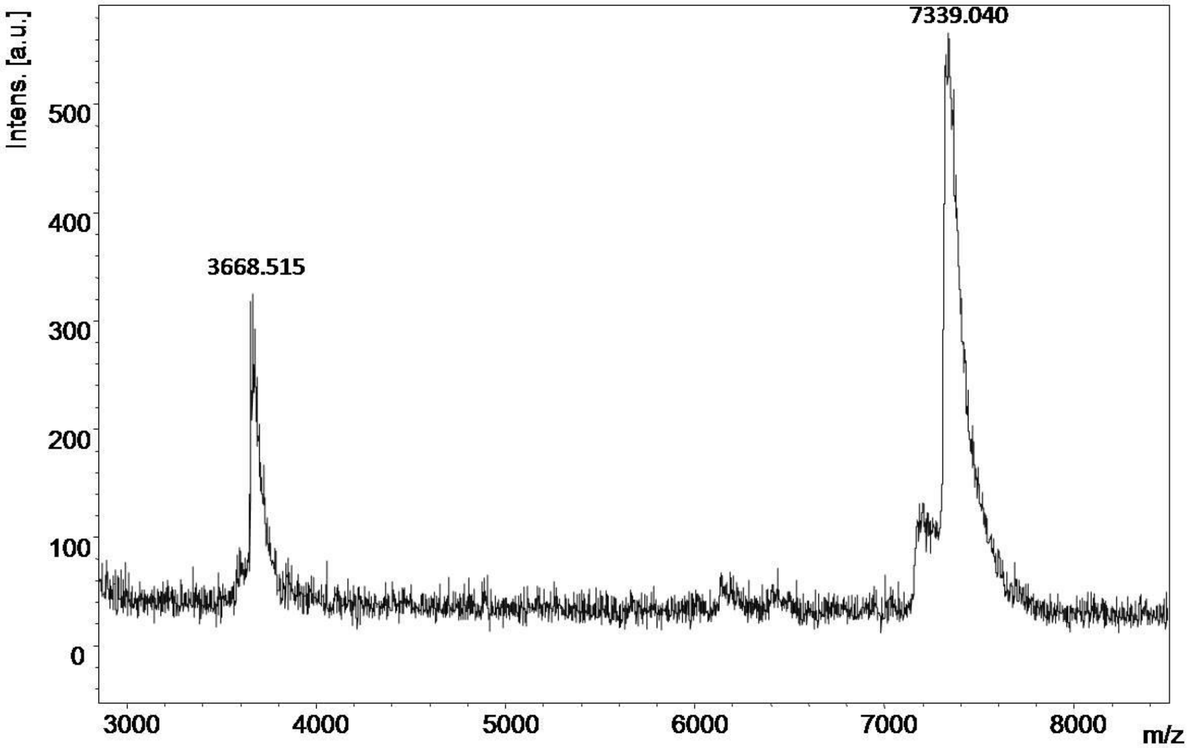

Supplement: File 1 — Experimental part. [file Beilstein_J_Org_Chem-10-1906-s001.pdf]
